# Within-Genome Evolution of REPINs: a New Family of Miniature Mobile DNA in Bacteria

**DOI:** 10.1371/journal.pgen.1002132

**Published:** 2011-06-16

**Authors:** Frederic Bertels, Paul B. Rainey

**Affiliations:** 1 New Zealand Institute for Advanced Study and Allan Wilson Centre for Molecular Ecology and Evolution, Massey University at Albany, Auckland, New Zealand; 2 Max Planck Institute for Evolutionary Biology, Plön, Germany; University of Toronto, Canada

## Abstract

Repetitive sequences are a conserved feature of many bacterial genomes. While first reported almost thirty years ago, and frequently exploited for genotyping purposes, little is known about their origin, maintenance, or processes affecting the dynamics of within-genome evolution. Here, beginning with analysis of the diversity and abundance of short oligonucleotide sequences in the genome of *Pseudomonas fluorescens* SBW25, we show that over-represented short sequences define three distinct groups (GI, GII, and GIII) of repetitive extragenic palindromic (REP) sequences. Patterns of REP distribution suggest that closely linked REP sequences form a functional replicative unit: REP doublets are over-represented, randomly distributed in extragenic space, and more highly conserved than singlets. In addition, doublets are organized as inverted repeats, which together with intervening spacer sequences are predicted to form hairpin structures in ssDNA or mRNA. We refer to these newly defined entities as REPINs (REP doublets forming hairpins) and identify short reads from population sequencing that reveal putative transposition intermediates. The proximal relationship between GI, GII, and GIII REPINs and specific REP-associated tyrosine transposases (RAYTs), combined with features of the putative transposition intermediate, suggests a mechanism for within-genome dissemination. Analysis of the distribution of REPs in a range of RAYT–containing bacterial genomes, including *Escherichia coli* K-12 and *Nostoc punctiforme*, show that REPINs are a widely distributed, but hitherto unrecognized, family of miniature non-autonomous mobile DNA.

## Author Summary

DNA sequences that copy themselves throughout genomes, and make no specific contribution to reproductive success, are by definition “selfish.” Such DNA is a feature of the genomes of all organisms and evident by virtue of its repetitive nature. In bacteria the predominant repetitive sequences are short (∼20 bp), extragenic, and palindromic. These so-called REP sequences may occur many hundreds of times per genome, but their origins and means of dissemination have been a longstanding mystery. We show that REPs are components of higher-order replicative entities termed REPINs, which are themselves thought to be derived from REP sequences that flanked an ancestral autonomous selfish element. In this ancestral state the REP sequences were likely to have been critical for the movement of the selfish element, but were devoid of any capacity to replicate independently. REPINs, on the other hand, have evolved to have a life of their own, albeit one that exploits—even enslaves—a genetic element upon which their existence depends. REPINs are the ultimate non-autonomous, super-streamlined, selfish element and are widespread among bacteria.

## Introduction

Short repetitive sequences are a feature of most genomes and have consequences for genome function and evolution [1], [2]. Often attributable to the proliferation of selfish elements [3], [4], short repeats also arise from amplification processes, such as replication slippage [5] and *via* selection on genome architecture [6]–[8].

Repetitive DNA in bacterial genomes is less prominent than in eukaryotes, nonetheless, an over abundance of short oligomers is a hallmark of almost every microbial genome [9]. Known generically as interspersed repetitive sequences, these elements have a history of exploitation as signatures of genetic diversity (e.g., [10]–[12]), but their evolution, maintenance and mechanism of within- and between-genome dissemination are poorly understood [9], [13]–[16].

Interspersed repetitive sequences fall into several broad groups each sharing short length (individual units range from ∼20 to ∼130 bp), extragenic placement, and palindromic structure [9], [17]. REPs (repetitive extragenic palindromic sequences) – also known as PUs (palindromic units) – range from ∼20 to ∼60 bp in length, possess an imperfect palindromic core, are widespread among bacteria, and occur hundreds of times per genome [13], [18]–[23]. While often existing as singlets, REPs also form a range of complex higher order structures termed BIMEs (bacterial interspersed mosaic elements) [14]. CRISPRs (clustered regularly interspaced short palindromic repeats) are a further, higher order composite of REP-like sequences that are formed from direct repeats of short (∼30 bp) palindromic sequences interspersed by similar size unique non-repeated DNA ([24]; reviewed in [25]). Recent work shows that the unique sequences are often phage derived and that CRISPRs, along with associated proteins, confer resistance to phage by targeting viral DNA [25], [26].

Non-autonomous DNA transposons form a more distinct family of repetitive sequences defined by their size (∼100 to ∼400 bp) and presence of terminal inverted repeats. Also known generically as MITEs (miniature inverted repeat transposable elements), non-autonomous transposons depend on transposase activity encoded by co-existing autonomous transposons for dissemination [4]. Identified initially in plants [27], where evidence of active transposition has been obtained [28], recent bioinformatic analyses suggest that they also occur in bacteria [29], [30]. For example, ERICs (enterobacterial repetitive intergenic consensus) – found in a range of enteric bacteria including *Escherichia coli*, *Salmonella* and *Yersinia* [31] – and NEMISs (*Neisseria* miniature insertion sequences) in pathogenic neisseriae [32] are thought to be non-autonomous transposons (MITEs).

Scenarios for the origins and functional significance of non-autonomous elements, and to a lesser extent CRISPRs, can be envisaged, but this is not so for the majority of short interspersed repetitive sequences. Nonetheless, studies of specific elements in particular genetic contexts have uncovered evidence of functional roles ranging from transcription termination and control of mRNA stability, to binding sites for DNA polymerase I (reviewed in [9]). However, the fact that the distribution and abundance of elements show substantial among-strain diversity [16], [22] suggests that the range of functional roles is incidental, arising from, for example, co-option or genetic accommodation [31].

Differences in the distribution and abundance of repetitive elements among closely related strains carries additional significance in that it suggests that the evolution of these elements is independent of the core genome. This is particularly apparent from comparisons of closely related strains. For example, *Pseudomonas fluorescens* isolates SBW25 and Pf0-1 are closely related and yet highly dissimilar in terms of the nature, abundance and distribution of interspersed repetitive elements [22], even, as we show here, at the level of REPs. While this may reflect unequal rates of element loss, an alternative possibility is independent acquisition. Implicit in this suggestion is the notion that repetitive elements are genetic parasites [13], [31], [33].

The idea that REPs are selfish elements is not new [13], [31], [33]; however, there is little evidence – either direct or indirect – to support such an assertion. Indeed, the small size of REPs makes a mechanism for autonomous replication difficult to envision, however, the recent discovery of a proximal association between REPs and IS*200*-like elements, termed RAYTs (REP-associated tyrosine transposases) [23], raises interesting possibilities and suggests shared ancestry between RAYTs and certain REP families.

Evolutionary approaches to the analysis of sequence motifs can be highly informative [34]. While there is a ready tendency to assume that motifs recognized by search algorithms have functional significance, this need not be so. Neutral evolutionary processes alone (nothing more than random chance) ensure that short sequences will occur multiple times within any given genome. Thus, before concluding functional significance, it is necessary to test the null hypothesis of chance. Should this hypothesis be rejected, then the conclusion that over-abundance of short sequences is attributable – at least in part – to natural selection is sound. Moreover, evidence for selection justifies the assumption of functional significance. A key issue, however, is the level of biological organization at which functionality has been selected. There are two distinct possibilities: short repeats may have evolved because of selective benefits conferred on the cell, but alternatively, they may deliver benefits at the level of the gene – more specifically, at the level of a genetic element, of which the repeat sequence is a component. Distinguishing between these two alternatives is possible, although not necessarily straightforward. Indeed, whereas on initial emergence, selection is likely to operate exclusively at one level, over time, it is likely to shift to encompass multiple levels [4], [16].

Here, we take a fresh and unbiased look at bacterial genome sequences in order to analyze the frequency and nature of short sequence repeats. Our approach is informed by evolutionary theory and begins free of assumptions regarding functional significance. Accordingly, the null hypothesis that short sequence repeats are no more frequent than expected by chance is the initial focus. We begin by interrogating the *P. fluorescens* SBW25 genome. Using suitable null models we show that over-abundant oligomers – which cannot be accounted for by chance alone – fall into three separate groups, each with characteristics typical of REPs. Highly significant differences in patterns of REP abundance and diversity between SBW25 and a second closely related *P. fluorescens* strain led us to question the hypothesis that the causes of REP diversity are linked to cellular function. This prompted a search for a replicative unit, which, based on patterns of REP distribution, we argue is a REP doublet. We refer to these entities as REPINs (REP doublets forming hairpins) and provide evidence from population sequencing for the existence of a putative transposition intermediate. Finally, extension to a range of RAYT-containing bacterial genomes including *E. coli* K-12 and *Nostoc punctiforme* indicate that REP sequences, organized as REPINs, define a class of hitherto unrecognized miniature non-autonomous mobile DNA.

## Results

### Oligonucleotide frequencies in *P. fluorescens* SBW25 and comparison to null models

**Figure 1:**
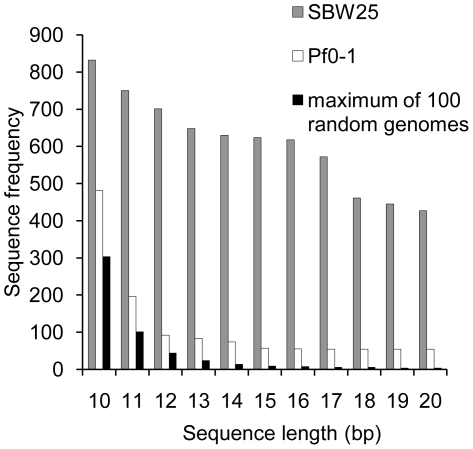
Frequency of common oligonucleotides in the genome of *P. fluorescens* SBW25. Data shows comparisons to both a random model, and to the closely related *P. fluorescens* Pf0-1 genome. The random model is based on 100 genomes generated with the same dinucleotide content, replication bias and length as the SBW25 genome. *P. fluorescens* Pf0-1 shares the same GC-content as SBW25 and has a highly similar dinucleotide content (Table S1); coding density differs by 1.7% and the genome length differs by 4%.

Defining repetitive DNA on the basis of short sequences ranging from 10–20 nucleotides is simple and can be done logically without invoking heuristics and approximations (for longer sequences exact repetitions are rare). Figure 1 shows that the *P. fluorescens* SBW25 genome harbors numerous repetitive sequences: the most common 10-mer occurs 832 times; the most common 20-mer occurs 427 times. While these numbers appear significant, it is possible that they are no more than expected by random chance. To test this hypothesis, 100 random genomes were generated, with the same dinucleotide content, replication bias and length, as the SBW25 genome. The frequency of the most abundant oligonucleotides was determined from both leading and lagging strands. Figure 1 shows that the most abundant 10-mer from the randomly generated genomes occurs 304 times. For longer sequence lengths this number rapidly decreases (four instances in the case of 20-mers): the number of repeats expected by chance alone is thus much lower than observed. In total, there are 108 different 10-mers and 14,351 different 20-mers that occur significantly more often in the *P. fluorescens* genome than the most abundant oligonucleotides from randomly generated genomes (*P*<0.01, Figure S1). While compelling evidence for the existence of over-representation of short sequences, gene duplications could in part account for these findings [35]. We therefore sought an alternative null model.

*P. fluorescens* Pf0-1, one of the closest relatives of SBW25, shares the same GC-content and has a highly similar dinucleotide content (Table S1); coding density differs by 1.7% and the genome length differs by 4% (6,722,539 bp for SBW25 and 6,438,405 bp for Pf0-1, [22]). The close similarity means that any bias in the representation of short sequences due to duplicative evolutionary processes, or other selective mechanisms, should be similar in both genomes.

As in SBW25, over-represented short sequences in Pf0-1 are more frequent than expected by chance (Figure 1), however, a considerable difference in short sequence frequency is apparent. The difference between SBW25 and Pf0-1 is greatest at a sequence length of 16, where the most abundant sequence in SBW25 occurs 618 times – over 11 times more frequently than the most abundant 16-mer in Pf0-1 (Figure S2). On the basis of comparisons to both the random null model and the Pf0-1 genome we deemed all SBW25 16-mers occurring more than 55 times (the frequency of the most abundant 16-mer in Pf0-1) to be over-represented. This led us to reject the null hypothesis that chance alone explains the occurrence of short repetitive sequences in the SBW25 genome. Accordingly, we attribute over-representation of oligonucleotides to selective processes.

### Short repetitive sequences in *P. fluorescens* SBW25 are synonymous with REPs

The collection of over-represented 16-mers together encompasses 96 different sequences; however, a cursory glance suggested that many share similarity. Using a grouping method designed to detect overlapping subsets of sequences (Methods and Figure S3), the 96 sequences were found to be members of just three separate sequence groups (GI, GII and GIII (Figure S4)), each containing an imperfect palindrome (the palindrome overlaps the most abundant 16-mer in GI and GII, but is part of the most abundant 16-mer in GIII (Table 1)). The most abundant 16-mers of each group together occur 1,067 times. The majority of these sequences are extragenic; only 14 16-mers overlap with genes. Together these data show that the three groups of 16-mers are over-represented in the SBW25 genome, contain an imperfect palindromic core and are primarily extragenic. Possessing the hallmarks of repetitive extragenic palindromic (REP) sequences, we conclude that the three groups of 16-mers are, for all intents and purposes, synonymous with REPs.

**Table 1:** Short repetitive sequence groups in the SBW25 genome.

Group^a^	Sequence^b^	Occurrences	Palindromic core^c^
I	GTGGGAGGGGGCTTGC	618	GGGGGCTTGCCCCC
II	GTGAGCGGGCTTGCCC	241	GCGGGCTTGCCCCGC
III	GAGGGAGCTTGCTCCC	208	GGGAGCTTGCTCCC

a 16-mers were sorted into three groups (GI, GII and GIII) using a grouping algorithm (Figure S3 and Figure S4).

b Sequence of the most common 16-mer from each group.

c Each GI, GII and GIII sequence either contains, or overlaps, an imperfect palindrome (the palindromic core).

### Determining REP sequence family size

In order to accommodate the possibility of related family members, we generated a pool of sequences that differed to GI, GII and GIII sequences by up to four bases. This generated 488,373 different 16-mers of which 1,861 were located in extragenic space. To define the proportion of false positives the search was repeated by interrogating randomly generated extragenic space (with the same dinucleotide content and length of each individual extragenic space) for matches to the 488,373 different 16-mers. This showed that 12% of all sequences with up to four substitutions are false positives (sequences unrelated to GI, GII or GIII). Repeating the analysis with the subset of sequences, which differ firstly by three and subsequently, two substitutions showed that 2% and 0.2% of matches are false positive, respectively. For two substitutions the false positive rate is low enough to conclude that the described repetitive sequence families consist of at least 1,422 members (Table 2). The precise number of members belonging to each of the GI, GII and GIII groups cannot be determined because with a degeneracy of two, some sequences fall into more than one group.

**Table 2:** Frequency of GI, GII, and GIII 16-mers in the extragenic space of the SBW25 genome.

	Number of occurrences	
Number of 16-mers^a^	Extragenic space	Randomly assembled extrangenic space^b^
0 substitutions(3 sequences)	1053	<0.01
1 substitution(147 sequences)	1249	0.13±0.33
2 substitutions(3,387 sequences)	1422	2.24±1.41
3 substitutions(48,707 sequences)	1560	31.18±5.18
4 substitutions(488,373 sequences)	1861	264.74±15.87

a In order to identify closely related members of each GI, GII and GIII sequence family extragenic space was searched for all possible sequences that differed by up to four substitutions. The number in brackets is the number of variant sequences: e.g., with no substitutions there are just the three sequences (Table 1); allowing one substitution there are 147 different sequences, and so forth. The number found in extragenic space was compared to a null (random) model based on randomly assembled extragenic space (see text).

b Data are means and standard deviation from 100 independent extragenic space randomizations.

### The distribution of REP sequences in the genome of SBW25

The selective causes for the prevalence of GI, GII and GIII sequences in the SBW25 genome are of considerable interest. Although implicit in many studies is the notion that REP-like sequences have evolved because of their selective benefit to the cell (as transcription binding sites, termination signals and the like [20], [36], [37]), it is also possible that selection has favored their evolution as a consequence of benefits delivered to a genetic (parasitic) element, of which the repeat sequence is a component. The highly significant differences in the frequency, nature and genomic location of short repetitive sequences in SBW25, compared to Pf0-1 make a compelling case for the latter.

If the prevalence of GI, GII and GIII sequences is a consequence of gene-level selection, then this implies the existence of a replicative entity – a genetic element that has the capacity to reproduce within the genome. The distribution of REP sequences is likely to provide some information. One way to quantify the distribution is to measure distances between neighboring REP sequences and compare these to distances between REPs generated by a null (random) model. If individual REPs are randomly distributed then this would suggest the individual REP as replicative unit. If the distance between adjacent REPs is non-random, then this may suggest the evolving entity is some higher order arrangement of REPs.

**Figure 2:**
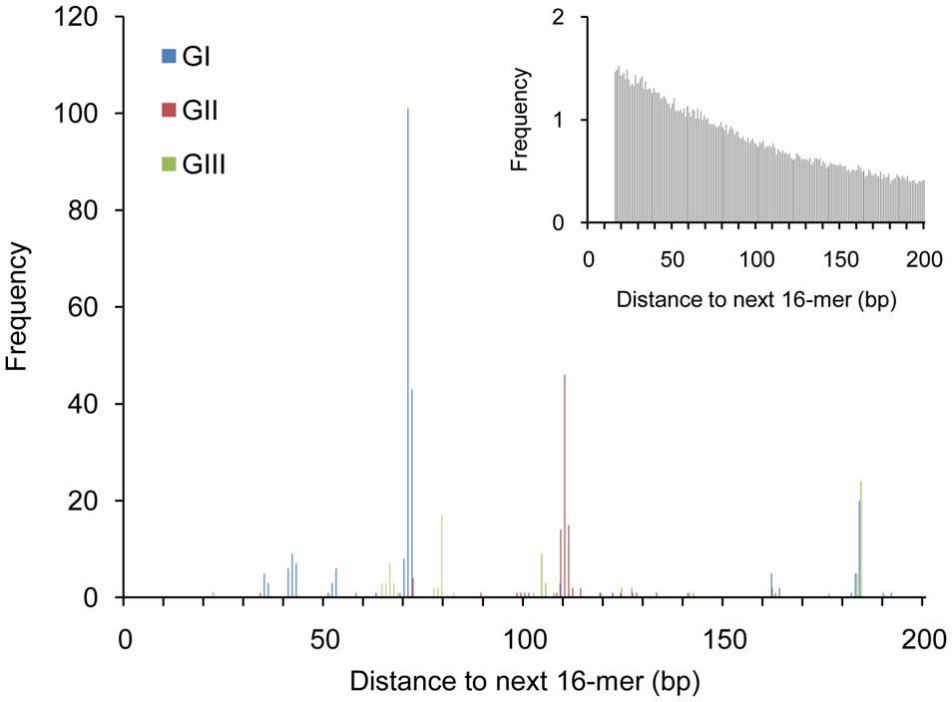
Frequency of next-neighbor distances for GI, GII, and GIII sequences in the genome of *P. fluorescens* SBW25. Data are next-neighbor distances for 1,053 GI, GII and GIII sequences in extragenic space, compared to a random model (inset). The peaks at 71 and 110 bp correspond to doublets of GI and GII sequences, respectively. The peak at 184 bp corresponds to GI–GIII tandem repeat clusters (see text). No significant deviation from the random model was noted for next-neighbor distances above 200 bp. The next-neighbor distances of 16-mers randomly assigned to extragenic space is the average of 10,000 simulations (inset).

To construct the null model, 1,053 (the number of invariant GI, GII and GIII sequences in extragenic space) non-overlapping 16 bp segments were positioned at random within the extragenic space of the SBW25 genome. This process was repeated 10,000 times and the average occurrence of the distance between neighboring elements calculated. Equivalent data for the 1,053 over-represented REPs is shown in Figure 2. A comparison between the two histograms reveals marked differences in the distributions of distances between next-neighbors. Most striking is the strong bias toward specific inter-element distances. This marked skew shows that REPs are not independently distributed and is suggestive of an underlying copying mechanism involving at least two REP sequences. Of note is the fact that doublets typically comprise pairs of identical GI, GII or GIII sequences and are rarely mixed (although some exceptions are discussed below) (Figure 2).

### The replicative unit

To explore the possibility that the replicative unit is an entity comprised of two REP elements (a REP doublet) we determined the number of singlets, doublets, triplets and higher order arrangements of REPs (REP clusters) by examining the 400 bp flanking either side of each REP for the presence of REP sequences (Figure S5). Once again, the results of this analysis were compared to the null (random) model used above.

According to the random model, 58% of all REP sequences are expected to occur as singlets, whereas data from SBW25 shows that just 18% are singlets. In contrast, 61% of all REPs are organized as doublets, which is significantly greater than the 17% expected by chance (Table 3). Interestingly, REP triplets are rarer than expected, whereas several higher order arrangements of REPs, including two sets of twelve (see below), are more frequent than expected (Table 3).

**Table 3:** Frequency of REP clusters within the SBW25 genome.

Cluster Size	Number of occurrences		*P*-Value	
	Observed^a^	Expected (random model)^b^	≤^c^	≥^d^
1	267	832±22.24	1	0
2	431	181.4±11.12	0	1
3	26	44.3±6.1	0.9998	0.0009
4	12	13.1±3.42	0.6658	0.4537
5	1	4.38±1.96	0.9893	0.0615
6	6	1.67±1.03	0.0070	0.9989
7	5	0.66±0.65	0.0007	0.9999
8	5	0.31±0.46	0	1
9	3	0.14±0.35	0.0006	1
10	0	0.07±0.25	1	0.9364
11	0	0.04±0.18	1	0.9658
12	2	0.02±0.14	0	1
**Sum**	1422	1421.76		

Data are the number of REPs occurring as clusters (from singlets to clusters of 12) in extragenic space compared to expectations from a null model based on the random assignment of 1,422 16-mers (to extragenic space) (see text).

a Observed occurrences from the SBW25 genome.

b Expected values (means and standard deviation) based on 10,000 simulations.

c The proportion of times the observed frequency was less than or equal to the expected value.

d The proportion of times the observed frequency was greater than or equal to the expected value.

The highly significant over-representation of REP doublets suggests that the doublet defines an appropriate replicative unit. If true, then the distribution of doublets across extragenic space should be unaffected by neighboring REP elements and should thus conform approximately to a null (random) model.

To test this hypothesis, random distributions of REP doublets over extragenic space were compared to actual REP clusters found in SBW25 (Table 4). However, because the distance between REPs (in the doublet conformation) varies (Figure 2), two random models were generated based on the two most common inter-REP spacings: 71 bp (a doublet of GI REPs) and 110 bp (a doublet of GII REPs). Simulations were based on the random assignment of 560 REP doublets (corresponding to the sum of REP clusters (of two or more) in Table 3) to extragenic space and were repeated 10,000 times. Although the two segments differ significantly in size, simulations for each family gave remarkably similar results (Table 4). Together these data show that the observed number resembles that predicted if the doublets are randomly distributed.

**Table 4:** Frequency of REP doublets within the SBW25 genome.

Segment length	Cluster size	Number of occurrences		*P*-Value	
		Observed^a^	Expected (random model)^b^	≤^c^	≥^d^
71 bp	2	457	434.76±12.9	0.0990	0.9144
	4	13	46.3±5.75	1	0
	6	11	7.69±2.6	0.0832	0.9575
	8	8	1.63±1	0.0001	1
	10	0	0.4±0.5	1	0.7323
	12	2	0.12±0.3	0.0023	0.9999
	14	0	0.03±0.18	1	0.9787
	16	0	0.01±0.1	1	0.9932
	18	0	0.002±0.06	1	0.9980
**Sum**		560	559.98		
110 bp	2	457	419.2±13	0.0167	0.9874
	4	13	49.1±5.9	1	0
	6	11	9.4±2.8	0.2112	0.8715
	8	8	2.2±1.2	0.0001	1
	10	0	0.7±0.6	1	0.6112
	12	2	0.2±0.4	0.0078	0.9998
	14	0	0.09±0.25	1	0.9553
	16	0	0.02±0.16	1	0.9834
	18	0	0.02±0.1	1	0.9944
**Sum**		560	560.07		

Data are the frequency of REP clusters (from doublets to cluster of 18 REPs) found in extragenic space compared to a null model based on the random assignment of 560×71 bp and 560×110 bp segments (to extragenic space). REP clusters containing an uneven number of REP sequences are included in the next lower cluster size (REP singlets are omitted).

a Observed occurrences from the SBW25 genome.

b Expected values (means and standard deviation) based on 10,000 simulations.

c The proportion of times the observed frequency was less than or equal to the expected value.

d The proportion of times the observed frequency was greater than or equal to the expected value.

**Figure 3:**
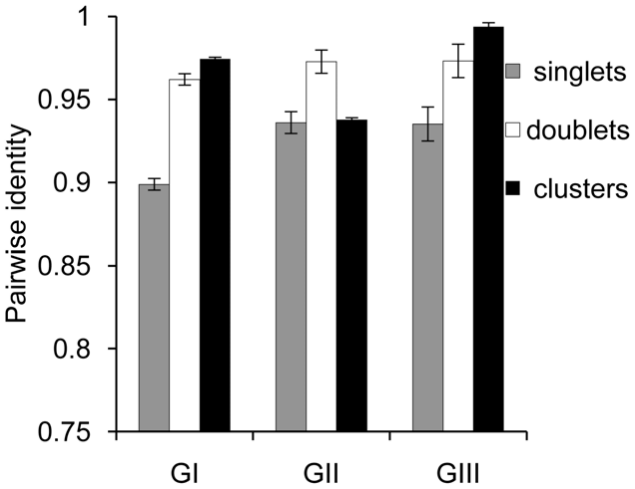
Average pairwise identity of REP sequences found in singlets, doublets, and clusters. Data are average pairwise identity of REPs found as singlets, doublets and clusters (clusters contain more than three REPs). Error bars show standard deviation. Statistical testing (jackknife) shows the average pairwise identity of 16-mers from REP doublets (and clusters for GI and GIII, *P*-value<1e-10) to be significantly greater than the average pairwise identity of 16-mers obtained from REP singlets: this is true for comparisons within each of the REP groups (*P*<1e-10 for GI; *P*<1e-8 for GII and GIII).

A further prediction concerns evolutionary processes affecting doublets vs. singlets. If REP doublets are the replicative unit, then singlets are likely to derive from doublets, either by decay (divergence) of the neighboring element, or by destruction of the doublet through insertion or deletion. In either case the REP singlet is expected to be non-functional (immobile) and thus subject to random genetic drift. REP doublets on the other hand – being (according to our hypothesis) functional and potentially mobile – are expected to be shaped by selection: genetic diversity of REP singlets should thus be greater than doublets. To test this hypothesis we extracted GI, GII and GIII sequences from the SBW25 genome plus all related sequences that varied by up to two positions. Since only two nucleotide differences distinguish GII and GIII sequences from a GI sequence, GII and GIII sequences were defined by two fixed (invariant) positions (GII: 2T, 6C; GIII: 6A, 13T). After extraction, sequences from each group were divided into a set of 16-mers obtained from singlets, a set of 16-mers from doublets and a set of 16-mers obtained from clusters (where a cluster contains three or more REPs). For all nine sequence groups (three from each GI, GII and GIII group) the pairwise identity was calculated (Figure 3, see Methods for details). The average pairwise identity of 16-mers obtained from REP doublets is significantly greater than the average pairwise identity of 16-mers obtained from REP singlets: this is true for comparisons within each of the REP groups (*P*<1e-10 for GI; *P*<1e-8 for GII and GIII).

**Figure 4:**
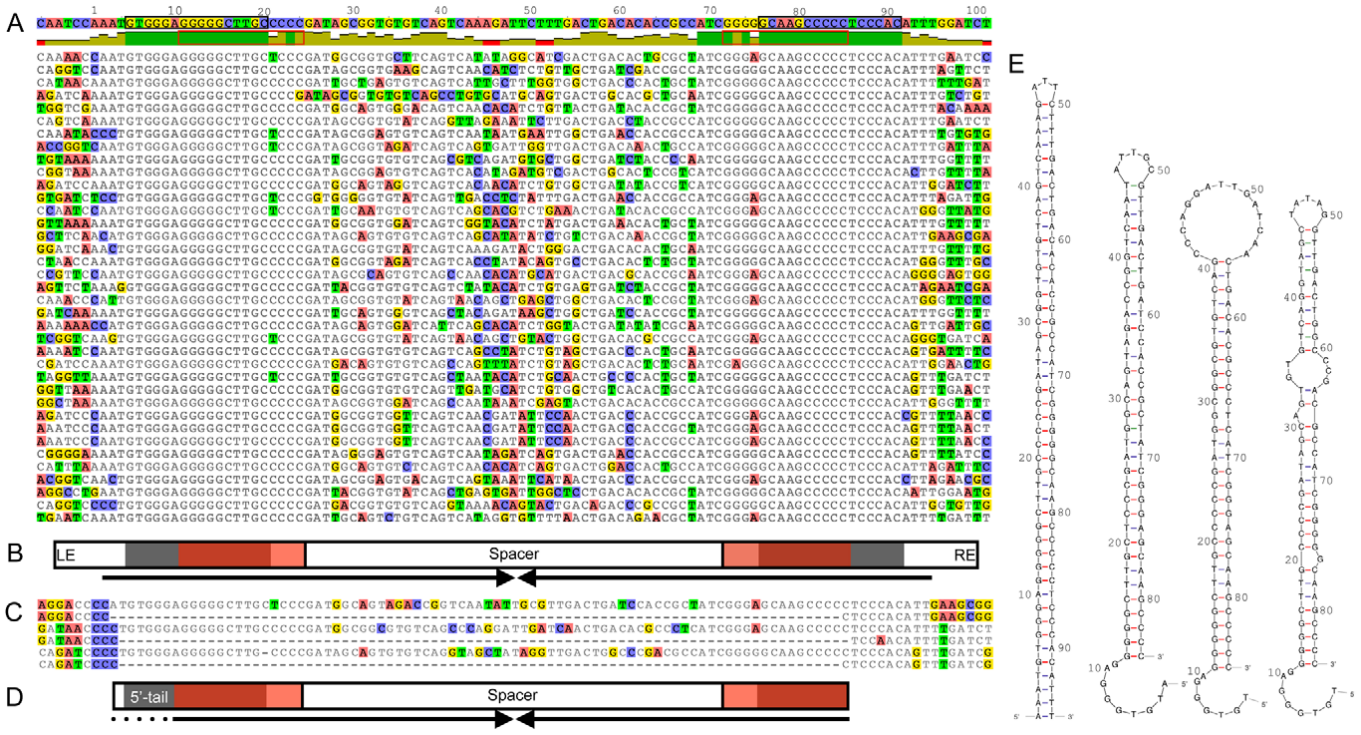
General organization and predicted secondary structure of REPINs. (*A*) Alignment of 101 GI REP doublets forming hairpins (REPINs) from SBW25 (37 are shown) shows a symmetrical (palindromic) organization comprised of two highly conserved regions separated by a spacer. Top line shows the consensus sequence followed by a graph displaying identity to the consensus (green denotes 100% identity). Two invariant regions of 16 bp are found in the left and right ends (LE, RE). These sequences are organized as inverted repeats and define the most abundant 16-mer in the SBW25 genome (black box). Each 16-mer overlaps a GI REP sequence (red box). (*B*) General REPIN features including LE and RE, each comprised of a highly conserved 16-mer (black) overlapping a REP sequence (red), with the two ends separated by a spacer. For a GI doublet the distance between the first residues of the two invariant 16-mers is 71 bp. Complementary bases permit formation of a hairpin structure (arrows). (*C*) Three excision events detected from Illumina sequencing reads reveal a putative transposition intermediate. Full-length sequences show three genomic regions located between 2,577,312–2,577,231, 3,857,520–3,857,439 and 5,683,545–5,683,624 bp on the SBW25 genome, each of which contains a REPIN. The partial sequences below each genomic region are Illumina reads from which the REPIN has been excised (see also Figure S6). (*D*) Cartoon of the excised region indicating putative transposition intermediate. Note the 5′-tail, which generates an asymmetrical sequence. (*E*) Secondary structure prediction for the consensus GI REPIN shows that the conserved bases on each side can pair resulting in a long hairpin (*E*, left). Predictions for transposition intermediates in the same order as the alignments in (*C*): the second, third and fourth hairpin correspond to the first, second and third alignment. The single stranded 5′-tail is free to pair with a complementary sequence.

Analysis of the organization of REP doublets shows that in the majority of cases, pairs of REPs (93% of all 430 REP doublets) – of either the GI, GII, or GIII types – are organized as two inverted REP sequences that overlap the most abundant 16-mer (Figure 4A and 4B). While the spacer region between REPs shows less conservation than evident in the REPs themselves, secondary structure predictions for ssDNA shows that the conserved bases on each side pair resulting in a hairpin (Figure 4E). Thus, while selection appears to favor highly conserved nucleotide arrangements for REP and adjacent sequences, the critical features of the intervening sequence would appear to be length, and capacity to form a hairpin. Indeed, compensatory changes on either side of the predicted hairpin are common (Figure 4A).

Finally, if our assertion that the doublet defines a replicative entity is correct, then evidence of movement could in principle come from population sequencing. To this end we interrogated 55,768,706 paired-end Illumina reads (36–76 bp long) obtained from sequencing DNA extracted from 5×10e9 SBW25 cells, for evidence of insertion and excision events. A total of 18 putative insertions were detected, however, the possibility of false positives could not be discounted. A similar search for excision events proved more profitable: three single reads were identified which mapped to three different locations on the genome, each corresponding to unique sequences flanking a GI REP doublet (Figure 4C and Figure S6). However, the expected doublet was absent from all sequence reads leading us to conclude that these sequences were from DNA molecules from which the doublet had excised. Additionally, we observed 200 individual sequence reads spanning a GII REP doublet indicating its excision from the entire population (Figure S6). That these events could result from machine and/or chemistry error is improbably low. Furthermore, a search for evidence of REP singlet deletions from the ∼56 million Illumina reads failed to find evidence of a single such event (see Methods).

Details of the three excised GI doublets are shown in Figure 4C and 4D. Of particular interest is the asymmetrical nature of the deleted sequence: in all instances it begins (in the left-hand (5′) end (Figure 4B)) at the start of the invariant sequence defined by the most conserved 16-mer and extends through the spacer region into the second REP sequence. However, rather than finish at the end of the conserved 16-mer, the deletion truncates at the 3′-end of the right-hand REP sequence, leaving the last ∼6 bp of invariant sequence intact (Figure 4C).

Secondary structure predictions show a hairpin structure with a 5′-single strand tail. Although the structures of the hairpins are not identical (due to differences in the sequence of the space region) the 5′-tail is a feature of the excised entity in all instances (Figure 4E). It is possible that the excised sequences define a putative transposition intermediate.

Together the above analyses implicate REP doublets as a unit of selection: a family of mobile DNA that has, until now, eluded recognition. Although REP doublets have previously been noted as one of many different higher order arrangements of REPs, they have not before been implicated as replicative entities [16]–[20]. Furthermore, in previous discussions of higher order arrangements it has been assumed that the singlet is the basic building block. In contrast, our data supports the view that REP singlets are defunct remnants of once functional REPINs. Because of their likely evolutionary relevance, a label that defines the replicative entity appears warranted. Henceforth we refer to REP doublets forming hairpins as REPINs.

### REPIN clusters

While the majority of REPINs exist as singlets, some higher order arrangements are apparent (above and Table 4). These are of two main types: those showing a distinctive ordering and those with no apparent structure.

REPINs occurring in ordered clusters are typically arranged as tandem repeats of nearly identical REPINs – including the flanking sequences (Figure S7). With 16 such clusters distributed throughout the genome, these arrays are the most common higher order arrangement of REPINs in SBW25. The largest cluster consists of four REPINs (plus an additional REP sequence) with a total length of over 700 bp.

**Figure 5:**
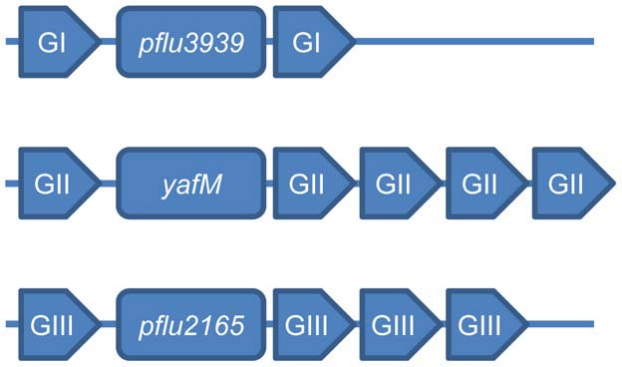
Proximity of GI, GII, and GIII REPIN clusters to RAYT genes in the *P. fluorescens* SBW25 genome. The RAYT genes in SBW25 are *pflu3939*, *yafM* and *pflu2165*.

Three higher order REPIN clusters are of particular note: one from each of the three distinctive REPIN groups (GI, GII and GIII) each located adjacent to one of the three recently identified REP-associate tyrosine transposases (RAYTs, [23]) (*pflu3939*, *pflu4255* and *pflu2165*). The fact that a different REPIN cluster is located beside each of the RAYTs, combined with the fact that REPINs (and REPs) in SBW25 come in three distinct flavors, raises the possibility that RAYTs are intimately linked to REPIN mobilization (Figure 5).

REPINs in clusters lacking obvious organization are found in five regions of the genome and typically consist of two unrelated REPINs. Close inspection suggests that these clusters are formed by insertion of REPINs into, or next to, existing REPINs.

### Tandemly repeated REP sequences

REPs also form higher order arrangements. These are of two distinct types: the first involves highly organized tandem arrays of GI and GIII REP sequences: GI REPs are separated from GIII REPs by 112 bp; GIII REPs are separated from GI REPs by 72 bp. Five such tandem arrays are located at ∼2 Mbp all of which are found in forward orientation, six are found ∼4 Mbp in reverse orientation (at a distance of ∼2 Mbp from the origin of replication). The two largest tandem arrays both contain 12 GI and GIII sequences, one found at ∼4.1 Mbp the other at ∼2.5 Mbp (Figure S8). These two arrays are almost identical copies of each other, but found in opposite orientations on opposite sides of the genome. The second type of tandemly organized REP sequences consists solely of evenly spaced GI sequences found at two positions in the genome. Similar to the GI–GIII tandem arrays one GI tandem array is found in forward and the other one in reverse orientation.

### REP sequence organization in other genomes

REPIN dissemination could occur *via* the exploitation of a functional transposase encoded separately within the genome. Non-autonomous DNA transposons (MITEs) do precisely this and typically consist of two inverted repeats. REPINs also consist of two inverted repeats (REP sequences) and, as mentioned above, may exploit the putative transposase encoded by RAYTs. If REP sequences in other genomes are components of REPINs – and disseminate *via* RAYT-encoded transposase activity – then, given the broad distributions of RAYTs [23], REPINs are likely to be a common feature of bacterial genomes; they are also likely to share common ancestry.

Although a fully comprehensive among-genome analysis is beyond the scope of this paper we nonetheless analyzed REP sequence clusters in a variety of genomes containing RAYTs. To this end REP sequences were selected from 18 different bacterial strains including all fully sequenced *Pseudomonas* genomes, the genomes of *E. coli* K-12 DH10B and *Salmonella enterica* serovar Paratyphi A AKU 12601 (chosen because of their significance for REP research) and the genomes of *Thioalkalivibrio* HL-EbGR7 and *N. punctiforme* PCC73102 (chosen because of their distant relation to *Pseudomonas*). A phylogenetic analysis of the RAYTs was firstly undertaken (Figure S9). Notably, RAYTs from these strains form two distinct evolutionary lineages with evidence of multiple independent introductions. For example, the genus *Pseudomonas* is separated into two sets of species defined by the presence of either ‘clade I’ or ‘clade II’ RAYTs. The genome of *Thioalkalivibrio* contains one clade I and one clade II RAYT. Several other genomes, in addition to SBW25, contain more than a single RAYT, but these almost never cluster. In fact the most closely related RAYTs are found in different genomes. Overall the distribution of RAYTs among distantly related organisms shows evidence of lateral gene transfer; however, at the species level, lateral gene transfer does not seem to occur frequently as evident by the fact that RAYT phylogeny is largely congruent with the relationship among species (Figure S9).

**Figure 6:**
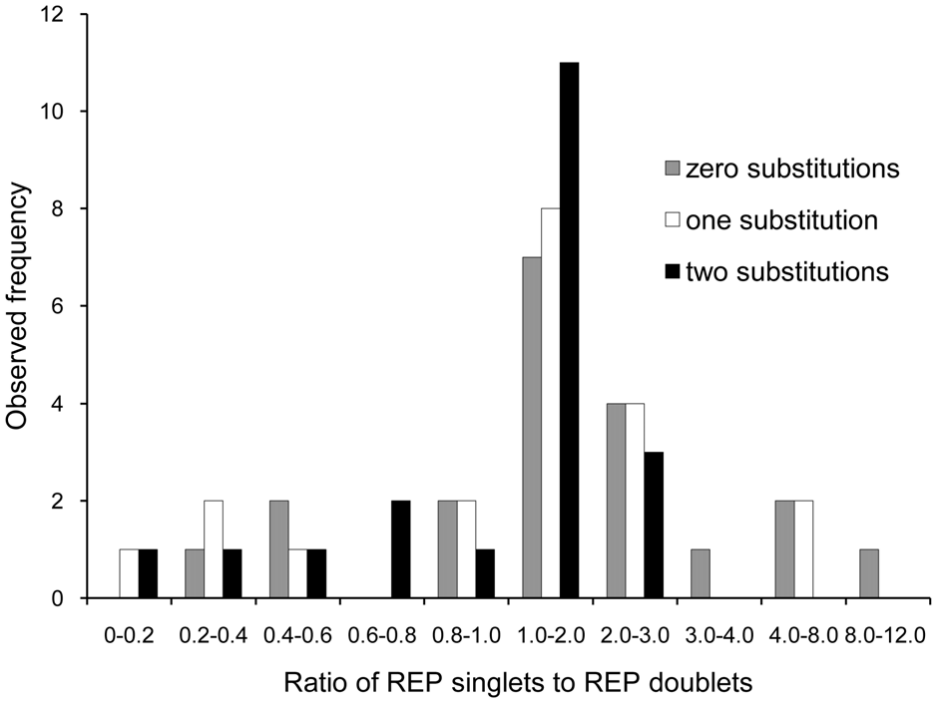
REP singlet to doublet ratios for REP sequences from bacterial genomes. Data are the most abundant 16-mers found within the flanking non-coding DNA of RAYT genes from 18 genomes. In order to include related 16-mers, a set of degenerate sequences was produced by allowing up to two substitutions per 16-mer.

Since REP sequences have been shown to be associated with RAYT genes (this work and [23]), we interrogated non-coding DNA flanking each RAYT for 16-mers that were repetitive, extragenic and palindromic, that is, are REPs. In each instance a REP was identified (Table S2). To test the hypothesis that REPs are organized as REPINs an analysis of the distribution of REPs was performed on each genome as described above (also see Methods) and included all REP sequences that differed from the consensus by up to two nucleotides. Results were expressed as the ratio of REP singlets to doublets, where ratios greater than two indicate that REPs occur predominantly as singlets. Ratios less than two mean that REPs occur predominantly as doublets. Figure 6 shows a histogram of singlet to doublet ratios for REP sequences associated with clade I RAYTs. Of the 20 REP sequence classes (one associated with each RAYT, some genomes contain more than one RAYT e.g., SBW25) 17 had singlet to doublet ratios of less than two, indicating that most REPs occur as doublets. The majority of doublets contained REPs as inverted pairs (Table S3) as expected of REPINs.

Our simple search method did not return conclusive results for clade II REP sequences. One possibility is that the REPIN structure in these genomes is less conserved. To this end we performed a secondary structure prediction on a sample of REP sequences. In all instances we found the general REPIN composition to hold (two inverted REP sequences separated by a short stretch of DNA and forming a hairpin, Figure S10), with the exception of REP sequences found in *P. stutzeri*: interestingly no REPINs were identified in this genome.

We also analyzed higher order arrangements for clade I REP sequences, but these were not present in all analyzed genomes. They were predominantly found in *P. syringae* and *P. fluorescens*, although two REP sequence classes were also detected in *P. putida* (Table S3). No correlation was found between the singlet to doublet ratio and cluster formation.

Taken together, the systematic cluster analysis of clade I REP sequences and secondary structure prediction of a selection of clade II REP sequences suggest that the organization of REP sequences into REPINs is a necessary condition for REP sequence distribution.

## Discussion

Short interspersed repetitive sequences are widely distributed in bacteria, but past studies have shed little light on their evolutionary origins. We began by examining the abundance and distribution of short sequences in *P. fluorescens* SBW25 and showed, by comparison against a random (null) model, and subsequently against Pf0-1, that short sequences are over-represented. Moreover, we found that short repetitive sequences fall into three distinct groups (GI, GII and GIII), each bearing characteristics typical of REP sequences, that is, they are repetitive, extragenic and palindromic.

In order to discount the possibility that REP sequences are the product of mutation pressure (a possibility already called into doubt by comparison to the random model) we took advantage of the closely related Pf0-1 genome. Comparisons using this null model – based upon a genome likely to have been shaped by similar underlying evolutionary processes – allowed us to emphatically reject the possibility that REP evolution can be explained by drift. Our data thus indicate natural selection as the primary driver of REP sequence evolution.

A critical issue is the nature of the entity upon which selection acts. Evidence that this entity comprises a doublet of REP sequences – a REP doublet forming a hairpin structure (REPIN) – came firstly from analysis of the distribution of REPs in extragenic space. The striking departure from a random model shown in Figure 2, along with clear bias toward specific distances between REPs, pointed to the REPIN as the replicative entity. The hypothesis was further tested by examining the distribution of REP doublets in extragenic space, by measuring nucleotide diversity in singlets versus doublets, and by analysis of the conserved features of REPINs. Finally, the existence of REPINs as actively mobile entities was bolstered through the discovery of four deletion events that may define putative transposition intermediates (Figure 4).

A previous analysis of the SBW25 genome using various repetitive DNA finding algorithms [22] revealed numerous repeat families. Two of these, the so named R0 and R2 repeats have characteristics similar to REPINs; indeed, a comparison (Table S4) shows a correspondence between REPINs and the R0 and R2 repeats. In general R0 repeats map to GI REPINs, while R2 repeats correspond to a mixture of both GII and GIII REPINs.

The mechanism by which REPINs are disseminated is a central, but unresolved issue. Recently, a hypothesis concerning REP sequence distribution was put forward [23]. The authors proposed that REP movement is effected by RAYTs – so named Y1 transposases – that are distantly related to the IS*200*/IS*605* family of insertion sequences. Integral to the transposition of IS*608* (a member of the IS*200*/IS*605* family) are two imperfect (REP-like) palindromes that flank either side of the insertion sequence and which are recognized by the transposase [38]. Whereas Nunvar et al. [23] suggested that REPs are moved by RAYTs, our data leads us to predict that it is the REPIN (and not the REP) that is mobilized *via* the RAYT: REPINs could be transposed by a RAYT dimer encoded in trans that recognizes the REP doublet. This mechanism would result in the strong conservation of the two REP sequences that define a REPIN (Figure S11).

The suggestion that RAYTs are integral to REPIN movement is given additional support by the discovery of excision events that appear to define the transposition intermediate. At first glance the footprints differ from expectations given that they do not encompass the full extent of the conserved REPIN (Figure 4B). However the asymmetrical nature of the putative intermediate is telling, particularly in light of the unusual mechanism of IS*608* transposition. IS*608* transposes *via* a single stranded intermediate and exploits singled stranded DNA at the replication fork; moreover, the intermediate involves pairing of asymmetric ends [38]–[40].

Assuming the excised DNA (Figure 4C and 4D) is a transposition intermediate then a key issue is re-establishment of the symmetrical REPIN. This could happen if the 5′-tail was involved in target recognition and paired with complementary sequence. In this regard it is of interest to note that the 5′-tail of the putative intermediate, which secondary structure predictions show is unlikely to form part of the hairpin (Figure 4E), is complementary to the 3′-end of the REPIN. It is possible that a recognition event involving pairing between complementary sequences, perhaps mediated *via* the RAYT, integrates back into DNA leading to the formation of a new REPIN. Although further insight requires molecular investigations, there exist a number of striking parallels with the mechanism of transposition of the IS*200*/IS*605* family of insertion sequences to which RAYTs – and their associated REPINs – are related.

While the argument for REPINs as replicative entities is supported by substantive data, REP singlets are nonetheless a notable feature of the SBW25 genome. Our data – particularly the significantly lower pairwise identity of REP singlets compared to REP doublets – suggests that these singlets are non-functional remnants of REPINs. But this does not explain why REP singlets are common. A close analysis of REP singletons reveals several possible routes for single REP sequences to emerge from REPINs. One possibility stems from limitations of our sequence search algorithms. When REPINs evolve neutrally successive acquisition of point mutations naturally leads to one REP becoming more decayed than the partner. If the less decayed REP is only just on the verge of recognition by our sequence search, then it is likely that the more decayed REP partner sequence will escape detection. A biologically plausible possibility is that singlets arise from insertion of DNA into REPINs. Indeed, earlier studies have noted that REP sequences are targets for certain insertion sequences [22], [41], [42]. REP singlets could also arise by deletion of the sequence between two REPs within a single REPIN leading to a long palindromic structure that contains only a single REP sequence: precisely such events can be seen in the genome of SBW25 (F. Bertels and P. B. Rainey, unpublished). A further possibility is that selection may act to preserve individual REP sequences because of specific functional consequences [16], [36].

A finding of note is the existence of several higher order arrangements of REPs and REPINs within the SBW25 genome, indeed, several such clusters occurred at a frequency above that expected from the null model (Table 3 and Table 4). Interestingly the majority of these clusters – at least those containing more than three REP sequences or REPINs – were arranged as highly ordered tandemly repeated units. This, combined with the fact that higher order arrangements were not found in all REPIN containing-genomes (Table S3), indicates a second mechanism for REP/REPIN cluster formation and suggests specific functional roles for these structures.

Extension of our analysis to a set of related (*Pseudomonas*) and unrelated (*E. coli*, *S. enterica*, *N. puctiforme* and *Thioalkalivibrio*) genomes each known to contain RAYTs showed that REPs in these bacteria are present in the immediate vicinity of RAYTs: moreover, in accord with predictions, these REPs are organized as REPINs. This finding greatly bolsters our conjecture that REPINs are a unit of selection, are RAYT associated, and widely distributed. In addition, the apparently general nature of the association between REPINs and RAYTs, combined with substantial diversity among the elements themselves, suggests that the diversity of REPINs (REPs) and RAYTs is a consequence of longstanding co-evolution between RAYTs and their respective REPINs.

The case for REPINs as widely distributed replicative entities is strong, but there remains much to be discovered, particularly regarding the mechanism of transposition, and the relationship between REPINs and RAYTs. A further unknown is the evolution of the entities themselves. One possibility is that REPINs are derived from the imperfect palindromic (REP) sequences flanking an ancestral IS*200*-like element – thus becoming non-autonomous elements [4] – but with a twist. Whereas non-autonomous elements exploit the transposase of extant transposons, the transposons they parasitize remain capable of autonomous replication. In contrast, RAYTs appear to be incapable of self-mobilization and exist as single copy entities (in those genomes harboring more than a single RAYT each RAYT is distinctive and present as just a single copy). This suggests that REPINs evolved a means of parasitizing an IS*200*-like ancestor that not only caused divergence of RAYTs from an IS*200*-like precursor, but did so in such a way as to enslave the RAYT. Just what keeps this association from extinction is among the more intriguing questions for future research, but suggests the existence of either an addiction system that ensures death of any cell that loses RAYT functionality, or a functional role for the RAYT in cell physiology that is somehow linked to REP function.

Finally, our evolutionary approach to the analysis of short repeats and discovery of REPINs and their associated RAYTs may prove useful for elucidating the origins of different kinds of short, repetitive, interspersed palindromic sequences such as NEMISs [32], ERICs [31] and small dispersed repeats (SDR) [43]. Indeed, REPINs themselves could conceivably constitute the building blocks for a range of more complex repetitive structures. For example, REPINs that incorporate DNA beneficial to a host bacterium are likely to have an advantage over standard REPINs. In this regard it is possible that CRISPRs [24] and related mosaic entities are derived from REPIN-like elements.

## Methods

### Generation of randomized genomes

100 genomes with the same dinucleotide content of the leading/lagging strand and length as the genome of *P. fluorescens* SBW25 were generated by randomly choosing nucleotides according to their occurrence probability based on the preceding nucleotide. To account for dinucleotide skew in the leading or lagging strand of the SBW25 genome, the dinucleotide content of the top strand was determined for the first half of the genome and of the bottom strand for the second half of the genome [22].

### Frequency determination of most abundant oligonucleotides

Sequence frequencies for all oligonucleotides of length 10 to 20 were determined using a sliding window with a step size of one for leading and lagging strand separately. The most abundant oligonucleotide for each sequence length was determined. This analysis was conducted for randomly generated genomes as well as for *P. fluorescens* SBW25 and Pf0-1.

### Grouping of highly abundant oligonucleotides in SBW25

All oligonucleotides of the chosen sequence length that occur more often in SBW25 than in Pf0-1 were ordered into groups using the following algorithm: 1, Select the most abundant 16-mer from the list of 16-mers that occur more frequently than the most abundant 16-mer in Pf0-1; 2, interrogate the SBW25 genome; 3, extract all occurrences including 20 bp of flanking DNA; 4, concatenate, separating each sequence by a vertical bar (a symbol that is not part of the genomic alphabet); 5, search all remaining 16-mers from the list against the generated string; 6, remove from the list of 16-mers all those sequences found within the generated string and place into the same group as the query; 7, repeat until the list of 16-mers is empty (Figure S3).

### Extending REP sequence groups and identifying the frequency of false positives

The genome was searched for related elements by introducing base pair substitutions into the most abundant sequence of each group to a maximum of four. The newly generated sequences, as well as the most abundant sequence of each group, were then used to interrogate the genome and the number of occurrences was counted. In order to determine the false positive rate, a simulation program was written to determine the number of sequences found in randomly generated extragenic space (with the same dinucleotide content).

### Distribution simulation

In order to produce a null model against which the observed next-neighbor distances could be compared, 1,053 segments of length 16 were randomly assigned to the extragenic space of SBW25. The simulation was repeated 10,000 times and for each simulation the distances to neighboring segments were determined. Additionally, the formation of clusters by GI, GII and GIII sequences with up to two mismatches (1,422 sequences) was measured. A cluster of REP sequences was defined as a group of REP sequences where each REP sequence has two neighboring REP sequences within the group that are separated by less than 400 bp (the next-neighbor distances showed no significant deviations from randomly expected distances above 400 bp) and a maximum of two REP elements that have only one neighbor within the group which is separated by less than 400 bp.

The same method was applied when distributing doublets randomly over the genome. Instead of 1,422 16 bp long segments, 560×71 bp and 560×110 bp long segments respectively, were randomly assigned. The number of REP doublets was determined by only counting doublets and clusters of doublets. For clusters that contain an odd number of REP sequences, only the even proportion was counted, thus excluding singlets.

### Singlet decay

To compare the rate of decay between REP singlets and REP sequences that are part of clusters, REP sequences were divided into their respective groups and then subdivided depending on whether they are found in clusters, or as singlets. In order to include related sequences, the 16-mers were allowed to vary at up to two positions. Since GI 16-mers differ from GII and GIII 16-mers by only two nucleotides, GII and GIII sequences also had to have two group-specific bases (GII: 2T, 6C; GIII: 6A, 13T).

The significance of the singlet decay data was tested using a permutation test. Nine different REP sequence pools were created. Three sequence pools for each sequence group, one of which contained REP singlets, one REP doublets and one greater REP cluster sequences. Two sequences were randomly drawn without replacement from a specific sequence pool and their pairwise identity (the number of sites that are identical between the two sequences divided by the total number of sites) was calculated. This procedure was repeated until the sequence pool was empty. The whole process was repeated 100,000 times for each sequence pool, resulting in the calculation of 100,000 average pairwise identities (mean). For GI sequences the maximum mean calculated for REP singlets never exceeded the minimum mean for REP sequences arranged as doublets. For GII and GIII sequences the maximum mean of REP singlets did exceed the minimum mean of REP sequences from doublets when more than 1,000 means were produced, hence the lower significance of 1e-8. Additionally, for GI and GIII sequences the maximum mean for singlets also never exceeds the minimum mean for clusters (*P*-value 1e-10). The average of the calculated means and the standard deviation are displayed in Figure 3.

### REP sequence selection in other genomes

Since REP sequences have been shown to be associated with RAYT genes [23], we looked for 16-mers that were repetitive, extragenic and palindromic in the non-coding DNA flanking RAYT genes. The most frequent 16-mers found within the flanking DNA were also part of or contained a palindrome and were found predominantly in extragenic space, thereby fulfilling all REP sequence prerequisites (Table S2). These 16-mers were then used for a subsequent cluster analysis (flanking clade I RAYTs) or a sample DNA secondary structure prediction (flanking clade II RAYTs).

### Bioinformatics and phylogenies

Blast searches were performed using NCBI Blast [44]. The genome was browsed using Artemis [45]. Inverted repeats were identified using Repeat Finder [46]. The multiple alignments in Figure 4 were displayed with Geneious [47] (due to the perfectly conserved distances between the 16-mers, the sequences were aligned after extraction from the genome, no alignment method was needed). DNA secondary structures were predicted using the mfold web server [48]. The RAYT phylogenetic tree was based on a translation alignment (ClustalW2 [49]) as implemented within Geneious [47]. The tree was constructed using a neighbor-joining [50] bootstrap analysis (1000 replicates) also embedded in Geneious.

### Genomes used in our analysis

*Pseudomonas fluorescens* SBW25 (NC_012660.1) [22]

*Pseudomonas fluorescens* Pf0-1 (NC_007492.2) [22]

*Pseudomonas fluorescens* Pf-5 (NC_004129.6) [51]

*Pseudomonas syringae phaseolicola* 1448A (NC_005773.3) [52]

*Pseudomonas syringae syringae* B728a (NC_007005.1) [53]

*Pseudomonas syringae tomato* DC3000 (NC_004578.1) [54]

*Pseudomonas entomophila* L48 (NC_008027.1) [55]

*Pseudomonas putida* W619 (NC_010501.1)

*Pseudomonas putida* KT2440 (NC_002947.3) [56]

*Pseudomonas putida* F1 (NC_009512.1)

*Pseudomonas putida* GB-1 (NC_010322.1)

*Pseudomonas aeruginosa* PAO1 (NC_002516.2) [57]

*Pseudomonas aeruginosa* PA7 (NC_009656.1) [58]

*Pseudomonas aeruginosa* LESB58 (NC_011770.1) [59]

*Pseudomonas mendocina* ymp (NC_009439.1)

*Pseudomonas stutzeri* A1501 (NC_009434.1) [60]

*Salmonella enterica* serovar Paratyphi *A* AKU_12601 (NC_011147.1) [61]

*Escherichia coli* K-12 DH10B (NC_010473.1) [62]

*Thioalkalivibrio* sp HL-EbGR7 (NC_011901.1)

*Nostoc punctiforme* PCC 73102 (NC_010628.1)

### Population sequencing

Pure genomic DNA was isolated from a single SBW25 colony using a combination of chloroform, CTAB and column (Qiagen DNeasy Blood & Tissue Kit) purification techniques. The genomic DNA was sheared to ∼400 bp and 76 bp paired-end were sequenced on two channels of an Illumina GA-II flowcell using standard protocols. Raw data were filtered to generate a set of sequences no less than 36 bp in length. After mapping short reads to the SBW25 genome using the Mosaik software suite (http://bioinformatics.bc.edu/marthlab/Mosaik), reads that could not be mapped were screened for REPIN excisions. The screening was accomplished in two steps: 1, for each REPIN present in the SBW25 genome 12 bp of the 5′ and 3′ flanking sequences were extracted; 2, since all reads are shorter than 76 bp, none of the extracted flanking sequences should occur within one read, hence reads containing both 5′ and 3′ REPIN flanking sequences contain an excision. Details of the sequences from which REPINs were excised are given in Figure S6.

### Testing for excision of REP singlets

In order to identify excisions of short palindromic sequences it was necessary to define a seed sequence. The GI and GII sequences described above do not overlap the palindromic region and hence are not suitable for this purpose (Table 1). We therefore used an 18-mer containing the palindrome of the GI REP as the seed sequence (GGGGGCTTGCCCCCTCCC). From this seed sequence we generated a set of 18-mers with up to five mismatches. These sequences matched a total of 1376 positions in the SBW25. This set of 1376 sequences encompassed all three GI, GII and GIII REP sequence groups and their relatives. In addition, to allow for the possibility of inexact excisions of palindromes, we allowed the excision to include three additional base pairs on each side of the seed sequence. Armed with this set of sequences we interrogated the ∼56 million Illumina-generated sequence reads for evidence of excision events.

## Supporting Information

Figure S1 Number of different oligonucleotides in the genome of *P. fluorescens* SBW25 that occur more often than the most frequent oligonucleotides from randomly assembled genomes.
(PDF)

Figure S2 Ratio between the most abundant oligonucleotides from SBW25 and Pf0-1.
(PDF)

Figure S3 Flowchart for grouping over-represented 16-mers. The algorithm sorts all 16-mers that occur more frequently in SBW25 than the most abundant 16-mer in Pf0-1 into groups.
(PDF)

Figure S4 Alignments of the most abundant sequence groups in SBW25. GI sequences are shown in (*A*), GII sequences in (*B*) and GIII sequences in (*C*). The consensus sequence contains the respective palindromic cores (framed in red). Numbers to the left of the alignment denote the frequency of the respective 16-mer (e.g. the first 16-mer in (*A*) GGGCTTGCTCCCGATG occurs 57 times). Colored nucleotides within the alignment denote differences to the consensus sequence.
(PDF)

Figure S5 Process of REP sequence cluster determination. REP sequences are blue boxes. Red arrows indicate a sequence length of 400 bp. The algorithm starts with the position of the first REP sequence (a) and adds it to cluster 1. It then checks the distance to the next REP sequence. The distance to REP sequence (b) is less than 400 bp, hence, the size of cluster 1 increases by one. The distance from (b) to the next REP sequence (c) is greater than 400 bp, therefore, the final size of cluster 1 is two and a new cluster of size one is created called cluster 2. The distance from REP sequence (c) to the next REP sequence is greater than 400 bp; hence, cluster 2 is closed.
(PDF)

Figure S6 Excision events detected in Illumina sequencing data. (*A*) Shows fastq formatted raw Illumina sequences for the excision events and their corresponding paired ends or ‘mates’. Quality scores are the last line of each fastq entry. (*B*) In all cases Read 1 matches to a position close to the corresponding Read 2 as expected for paired end reads. The alignments show the match between the sequence reads (second line in the alignment) and the SBW25 genome (first line in the alignment). Colored nucleotides show differences between genome and sequence read. Secondary structure predictions of the excised sequences are shown on the right. For the fourth excision a total of 200 sequence reads were found showing the same event, indicating that the entire REPIN was excised from the genome.
(PDF)

Figure S7 Schematic representation of a typical tandemly repeated REPIN cluster. The cluster comprises two tandem repeat units. Each unit consists of a 5′ flanking sequence (f1) followed by a REPIN and ends with a second shorter flanking sequence (f2). The two units are usually separated by a short stretch of DNA that is not repeated.
(PDF)

Figure S8 Approximate positions of the tandem repeat clusters in the genome of SBW25. The tandem repeats are formed by sequences from GI and GIII. The gray and black arrows indicate different module lengths.
(PDF)

Figure S9 RAYT neighbor joining tree. Two distinct phylogenetic groups are present (Clade I and Clade II). The tree is based on a translated nucleotide alignment. The first part of the branch tip description denotes the gene name and the second part the name of the host organism.
(PDF)

Figure S10 REPIN secondary structures found in different genomes predicted by the mfold web server (http://mfold.rna.albany.edu/). Red bars show palindromic parts of the structure. The yellow box indicates the most abundant 16-mer found in the non-coding flanking DNA of the respective RAYT. The GI consensus sequence from *Pseudomonas fluorescens* SBW25 is the only REPIN shown from RAYT clade I (Figure 4), all other REPINs are associated to RAYTs from clade II.
(PDF)

Figure S11 Two different REPIN folds and their potential susceptibility for transposition by a RAYT dimer. According to our hypothesis the more stable hairpin structure formed by REPINs (left) is unlikely to be recognized by RAYTs and may be a mechanism to reduce the frequency of transposition within the genome. In contrast, the less stable “clover” configuration (right) is likely to be recognized in an *IS200* like manner and may lead to the excision of an asymmetric transposition intermediate.
(PDF)

Table S1 Dinucleotide frequencies in *P. fluorescens* Pf0-1 and SBW25.
(PDF)

Table S2 Short sequence composition of the non-coding DNA flanking RAYTs.
(PDF)

Table S3 Details concerning the analysis of REP sequences in other bacterial genomes.
(XLSX)

Table S4 Correlation between REPINs and repeat families previously detected in SBW25.
(PDF)
