# Supplementary figures and images for: Within-Genome Evolution of REPINs: a New Family of Miniature Mobile DNA in Bacteria
Source: PLoS Genet. 2011 Jun 16;7(6):e1002132. doi: 10.1371/journal.pgen.1002132 (PMC3116915; doi:10.1371/journal.pgen.1002132)

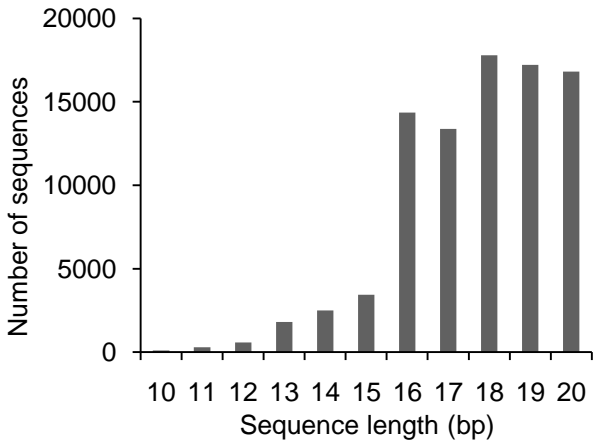

Supplement: Figure S1 — Number of different oligonucleotides in the genome of P. fluorescens SBW25 that occur more often than the most frequent oligonucleotides from randomly assembled genomes. (PDF) [file pgen.1002132.s001.pdf]

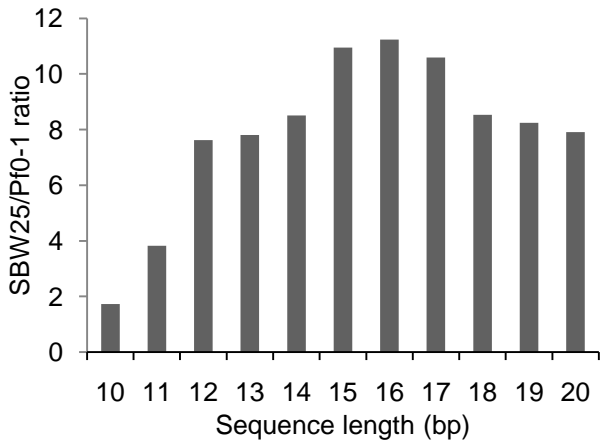

Supplement: Figure S2 — Ratio between the most abundant oligonucleotides from SBW25 and Pf0-1. (PDF) [file pgen.1002132.s002.pdf]

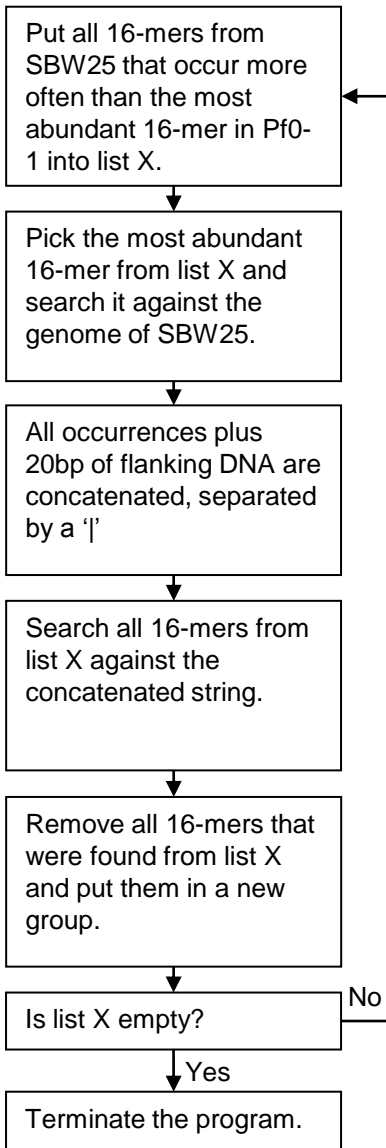

Supplement: Figure S3 — Flowchart for grouping over-represented 16-mers. The algorithm sorts all 16-mers that occur more frequently in SBW25 than the most abundant 16-mer in Pf0-1 into groups. (PDF) [file pgen.1002132.s003.pdf]

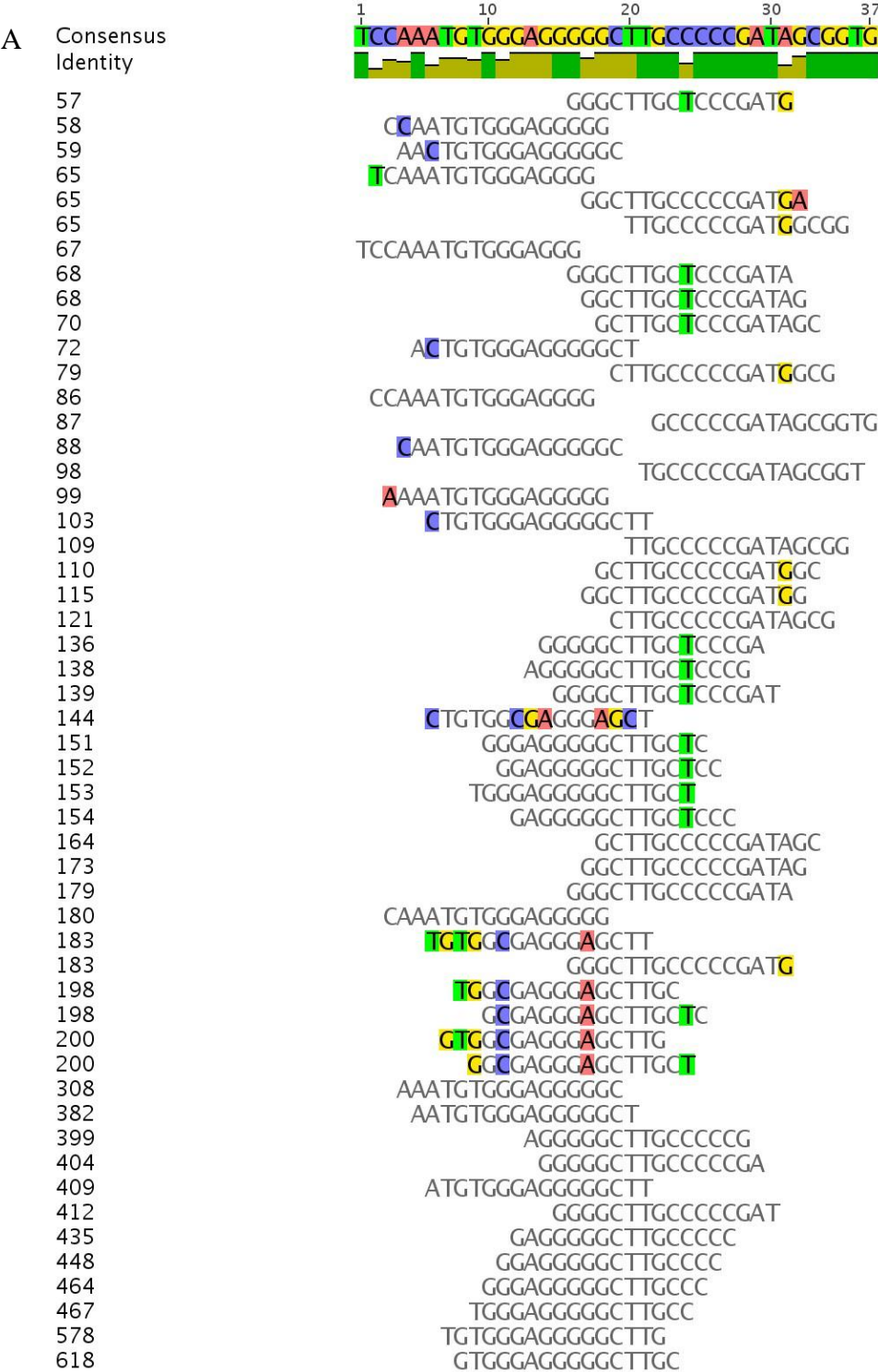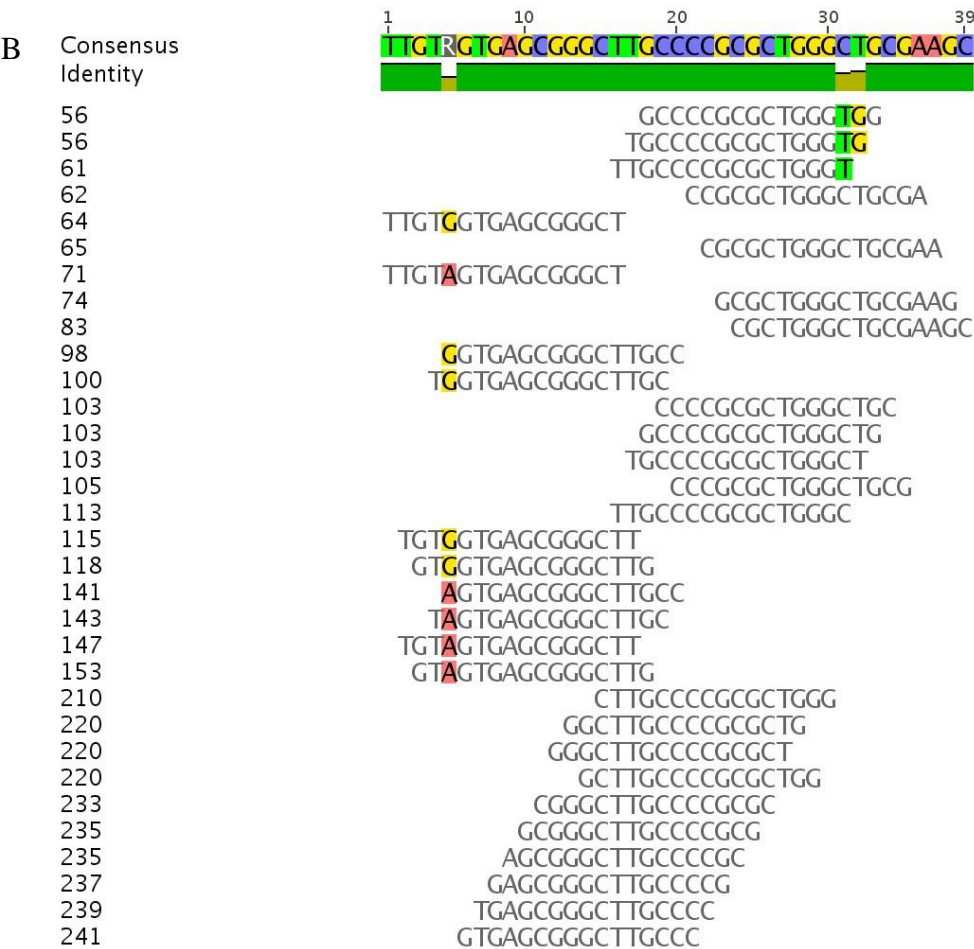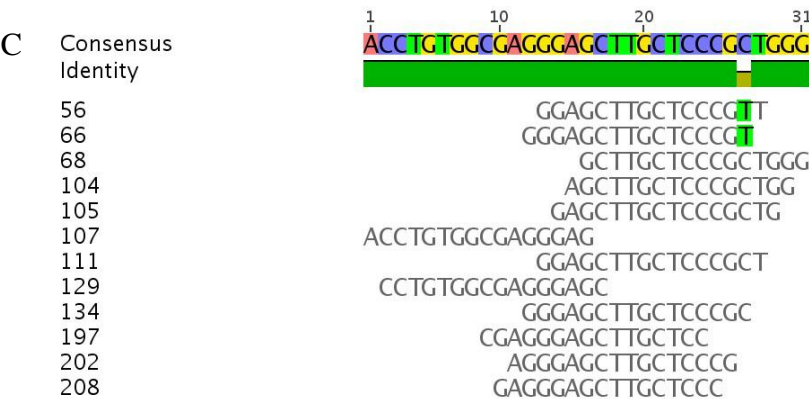

Supplement: Figure S4 — Alignments of the most abundant sequence groups in SBW25. GI sequences are shown in (A), GII sequences in (B) and GIII sequences in (C). The consensus sequence contains the respective palindromic cores (framed in red). Numbers to the left of the alignment denote the frequency of the respective 16-mer (e.g. the first 16-mer in (A) GGGCTTGCTCCCGATG occurs 57 times). Colored nucleotides within the alignment denote differences to the consensus sequence. (PDF) [file pgen.1002132.s004.pdf]

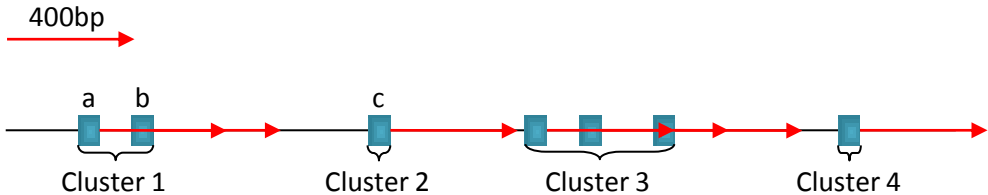

Supplement: Figure S5 — Process of REP sequence cluster determination. REP sequences are blue boxes. Red arrows indicate a sequence length of 400 bp. The algorithm starts with the position of the first REP sequence (a) and adds it to cluster 1. It then checks the distance to the next REP sequence. The distance to REP sequence (b) is less than 400 bp, hence, the size of cluster 1 increases by one. The distance from (b) to the next REP sequence (c) is greater than 400 bp, therefore, the final size of cluster 1 is two and a new cluster of size one is created called cluster 2. The distance from REP sequence (c) to the next REP sequence is greater than 400 bp; hence, cluster 2 is closed. (PDF) [file pgen.1002132.s005.pdf]

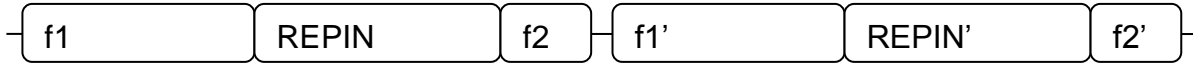

Supplement: Figure S7 — Schematic representation of a typical tandemly repeated REPIN cluster. The cluster comprises two tandem repeat units. Each unit consists of a 5′ flanking sequence (f1) followed by a REPIN and ends with a second shorter flanking sequence (f2). The two units are usually separated by a short stretch of DNA that is not repeated. (PDF) [file pgen.1002132.s007.pdf]

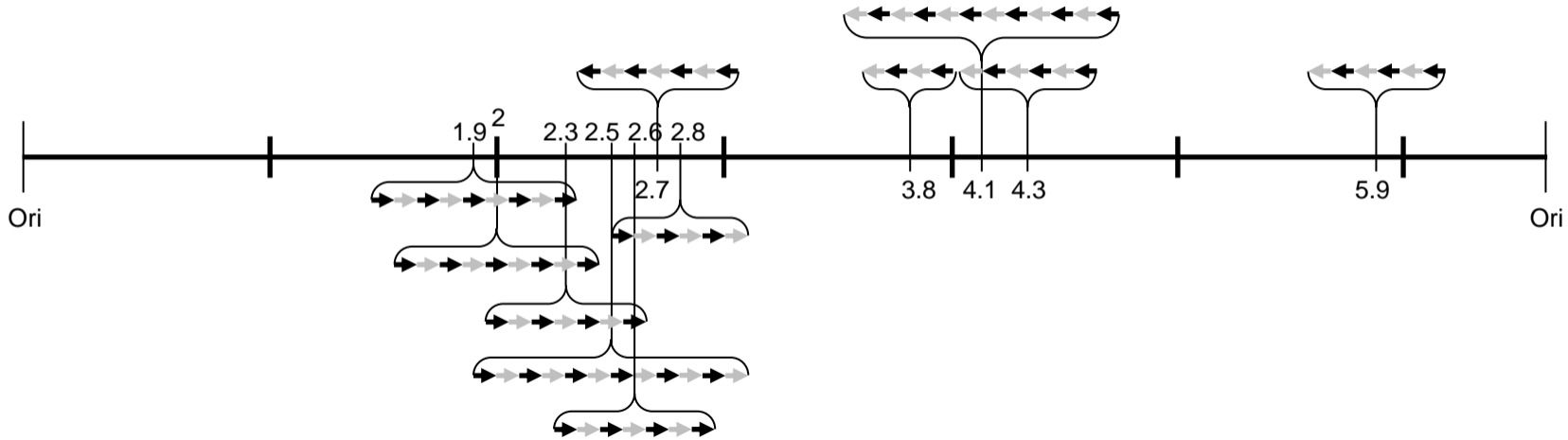

Supplement: Figure S8 — Approximate positions of the tandem repeat clusters in the genome of SBW25. The tandem repeats are formed by sequences from GI and GIII. The gray and black arrows indicate different module lengths. (PDF) [file pgen.1002132.s008.pdf]

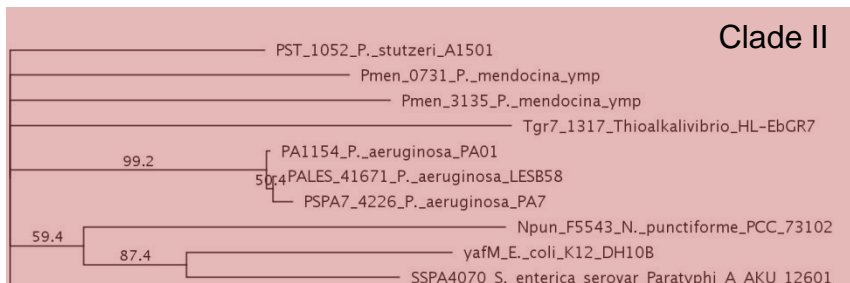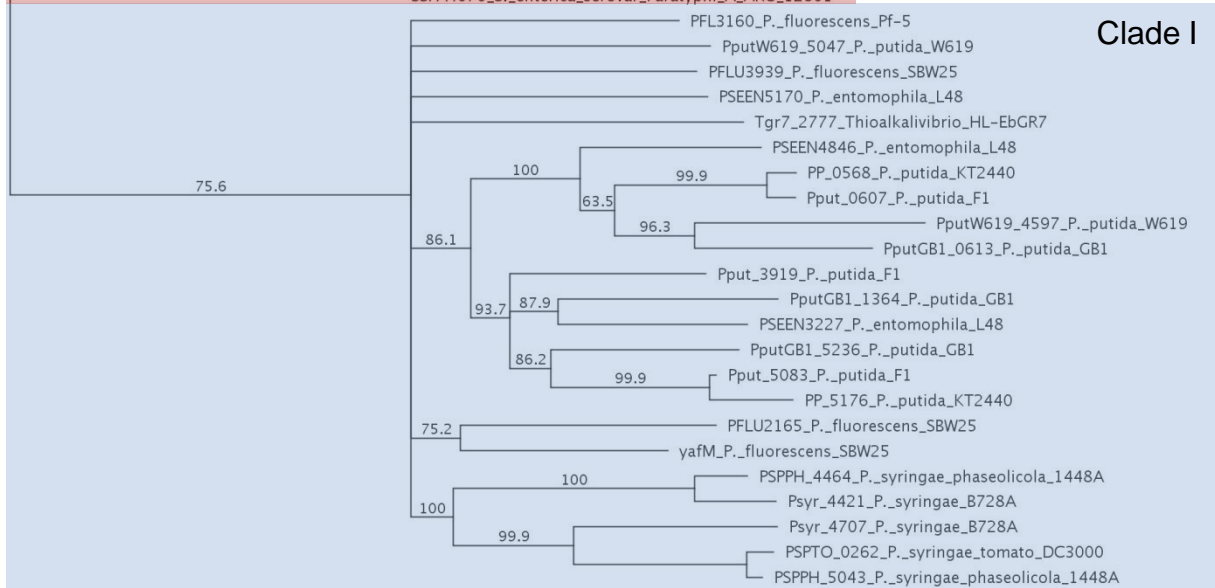

0.2

Supplement: Figure S9 — RAYT neighbor joining tree. Two distinct phylogenetic groups are present (Clade I and Clade II). The tree is based on a translated nucleotide alignment. The first part of the branch tip description denotes the gene name and the second part the name of the host organism. (PDF) [file pgen.1002132.s009.pdf]

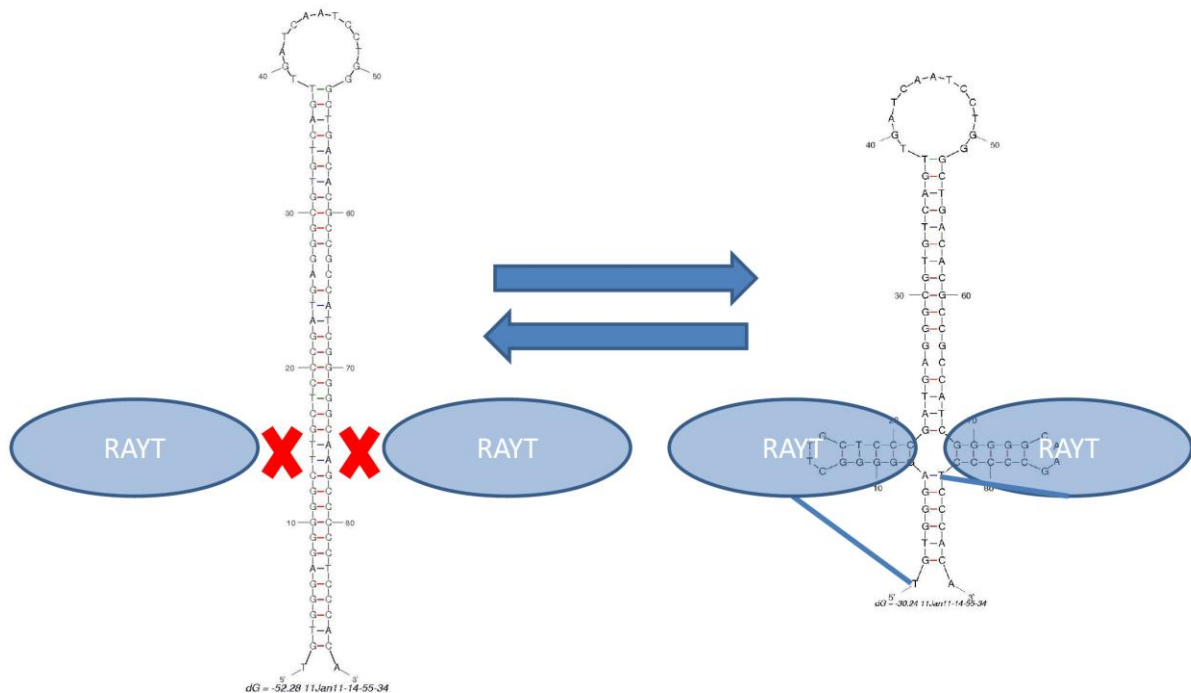

Supplement: Figure S11 — Two different REPIN folds and their potential susceptibility for transposition by a RAYT dimer. According to our hypothesis the more stable hairpin structure formed by REPINs (left) is unlikely to be recognized by RAYTs and may be a mechanism to reduce the frequency of transposition within the genome. In contrast, the less stable “clover” configuration (right) is likely to be recognized in an IS200 like manner and may lead to the excision of an asymmetric transposition intermediate. (PDF) [file pgen.1002132.s011.pdf]
