# Supplementary material for: Within-Genome Evolution of REPINs: a New Family of Miniature Mobile DNA in Bacteria
Source: PLoS Genet. 2011 Jun 16;7(6):e1002132. doi: 10.1371/journal.pgen.1002132 (PMC3116915; doi:10.1371/journal.pgen.1002132)

A

```
Read 1
@2_21_784_925
CGGCAGAGGTGATACTGGATGCGGTGGAGAGTGATGTGCTGGTGCTCAAGCCGCAGGCGTTGATGGATCAGCTCGN
+
AA?@BB<@@=;>@A1>?>@B>1=8..71<49'@4:>6A=;BA;A4>42?;,5=566####################
Read 2
@2_21_784_925
TTCCAACTGACAACGGATAACCCCTCCAACATTTTGATCTCCATCGTTCATAAGGTTGGATCAGGGCTTGNTNNNN
+
BC;BBCBBCCABC:BBBBBB?B>AB>BAB7B>ABBBABAB@?BB:C=B(>B==BA4@><@>9@<A###########
Read 1
@1_82_1308_1969
TACTACAAGGTCAACGACAAAGTGCGCCTGAACCTCGACGTGAAGAACCTGTTCAACCGCGAGTATGAAGAACGCG
+
6@AABCA>B>>;@?8>?@?A<4;>B<9?@8;A7)=B>A:B6BA@>B:'03>-<:>>?-<B@?A?<>=6@A53&7;@
Read 2
@1_82_1308_1969
ATACCCAGGACCCCCTCCCACATTGAAGCGGTGTACGCCGTCATAGCGTGTAGGCGAATGCGATGTGGACGGGCAN
+
AAAA99>A5>5:5?5@3<5>=AB@CB@:<A@/=7?99773&1,#################################
Read 1
@2_70_1540_677
GACATAACCACCAATCACCACAAAGGCCGTAACAGCGTCACCTGACACAACGCCGATCAAACTGTGGGAGGGGGAT
+
BCBCCCCBBCBBCCCCBBBCBBB?=AA;B?BB>BC@AABCBAB@BBCBBBBBBB@@=?=>@/BA<;AB@B;A@A29
Read 2
@2_70_1540_677
ATTATTGTGATTGCACCGTTTGGATATTCACGCAGATCCCCCTCCCACAGTTTGATCGGCGTTGTGTCAGGTGAAG
+
BCCCCCBCBBCCCBCBCBBCCCCCBBCBBBACACBCA@A@>@B@A@A=A@8=A@@=6?>AB@;;;:==><@/B9##
Read 1
@1_13_1051_777
CTGTCACCACACGCACAGGTGACGGGTGGTCGAGAGTAAAATCGTTCGCAAGCTACTTATCTATTTGCGACGCGCA
+
BA@A<A;A@:??A?6<9:/7;>?9'3,'82;A9?=A4177>>9@A?9=:4>207?5<<@?5;//67/33<@#####
Read 2
@1_13_1051_777
GCGGGCAGCGCAGTAGAGTGTTGAAGACTGTGTAGTGAGCGCCGGAGCGAAAAGCGCCACCTTTTGGCGCGGTGNN
+
B9AB9==@'@67BBB?:B>AABB6BB<16;2>,=;3.:(,B16A==;,5:366#######################
```

B

Read 1 @2_21_784_925 matches to region 3857305 – 3857360 (1 – 70) in the SBW25 genome.

Read 2 @2_21_784_925:

```
                 1        10        20        30        40        50        60        70
3857544..3857393 TTCCAACTGACAACGGATAACCCCTGTGGGAGGGGGCTTGCCCCCGATGGCGGCGTGTCAGCCCAGGATTGATCAA
2_21_784_925     TTCCAACTGACAACGGATAACCCC----------------------------------------------------

                    80        90        100       110       120       130       140       152
3857544..3857393 CTGACACGCCCTCATCGGGAGCAAGCCCCCTCCCACATTTTGATCTCCATCGTTCATAAGGTTGGATCAGGGCTTG
2_21_784_925     ------------------------------TCCAACATTTTGATCTCCATCGTTCATAAGGTTGGATCAGGGCTTG
```

Read 1 @1_82_1308_1969 matches to region 2577058 – 2577129 (1 – 72) in the SBW25 genome.

Read 2 @1_82_1308_1969:

```
                 1        10        20        30        40        50        60
2577326..2577202 ATACCCAGGACCCCATGTGGGAGGGGGCTTGCTCCCGATGGCAGTAGACCGGTCAATATTGCG
1_82_1308_1969   ATACCCAGGACCCC-------------------------------------------------

                       70        80        90        100       110       120  125
2577326..2577202 TTGACTGATCCACCGCTATCGGGAGCAAGCCCCCTCCCACATTGAAGCGGTGTACGCCGTCA
1_82_1308_1969   ---------------------------------CTCCCACATTGAAGCGGTGTACGCCGTCA
```

Read 2 @2_70_1540_677 matches to region 790520 – 790547 (1 – 28) and 5683675 – 5683621 (20 – 74) in the SBW25 genome.

Read 1 @2_70_1540_677:

```
                 1        10        20        30        40        50        60        70
5683504..5683657 ATTATTGTGATTGCACCGTTTGGATATTCACGCAGATCCCCTGTGGGAGGGGGCTTGCCCCGATAGCAGTGTGTCAG
2_70_1540_677    ATTATTGTGATTGCACCGTTTGGATATTCACGCAGATCCCC------------------------------------

                    80        90        100       110       120       130       140       150 154
5683504..5683657 GTAGCTATAGGTTGACTGGCCCGACGCCATCGGGGGCAAGCCCCCTCCCACAGTTTGATCGGCGTTGTGTCAGGTGA
2_70_1540_677    --------------------------------------------CTCCCACAGTTTGATCGGCGTTGTGTCAGGTGA
```

Read 1 @1_13_1051_777 matches to region 6222912 – 6222986 (1 – 75).

Read 2 @1_13_1051_777:

```
                 1        10        20        30        40        50        60        70
6223184..6222982 GCGGTCAGCGCAGTAGAGTGTTGAAGACTGTGTAGTGAGCGGGCTTGCCCCGCGCTGGGGGGCGAAGCCGCCCCAAT
1_13_1051_777    GCGGGCAGCGCAGTAGAGTGTTGAAGACTGTGT--------------------------------------------

                    80        90        100       110       120       130       140       150
6223184..6222982 AAAGGCACCACATAGTTTCAGGTAAAGCCGAGTCGCCTGATTTTGGGGCGGCTTCGCCCCCCAGCGCGGGGCAAGCC
1_13_1051_777    -----------------------------------------------------------------------------

                      160       170       180       190       200 203
6223184..6222982 CGCTCACTACAAGTGAGCGCCGGAGCGAAAAGCGCCACCTTTTGGCGCG
1_13_1051_777    -----------AGTGAGCGCCGGAGCGAAAAGCGCCACCTTTTGGCGCG
```

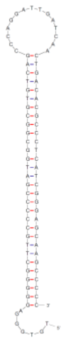

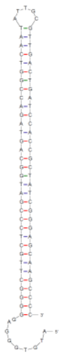

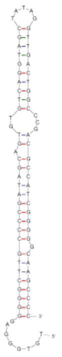

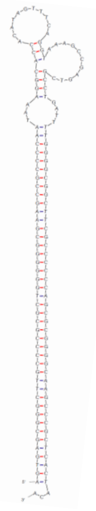

Supplement: Figure S6 — Excision events detected in Illumina sequencing data. (A) Shows fastq formatted raw Illumina sequences for the excision events and their corresponding paired ends or ‘mates’. Quality scores are the last line of each fastq entry. (B) In all cases Read 1 matches to a position close to the corresponding Read 2 as expected for paired end reads. The alignments show the match between the sequence reads (second line in the alignment) and the SBW25 genome (first line in the alignment). Colored nucleotides show differences between genome and sequence read. Secondary structure predictions of the excised sequences are shown on the right. For the fourth excision a total of 200 sequence reads were found showing the same event, indicating that the entire REPIN was excised from the genome. (PDF) [file pgen.1002132.s006.pdf]
