# Supplementary material for: Within-Genome Evolution of REPINs: a New Family of Miniature Mobile DNA in Bacteria
Source: PLoS Genet. 2011 Jun 16;7(6):e1002132. doi: 10.1371/journal.pgen.1002132 (PMC3116915; doi:10.1371/journal.pgen.1002132)
Supplement: Figure S10 — REPIN secondary structures found in different genomes predicted by the mfold web server (http://mfold.rna.albany.edu/). Red bars show palindromic parts of the structure. The yellow box indicates the most abundant 16-mer found in the non-coding flanking DNA of the respective RAYT. The GI consensus sequence from Pseudomonas fluorescens SBW25 is the only REPIN shown from RAYT clade I (Figure 4), all other REPINs are associated to RAYTs from clade II. (PDF) [file pgen.1002132.s010.pdf]

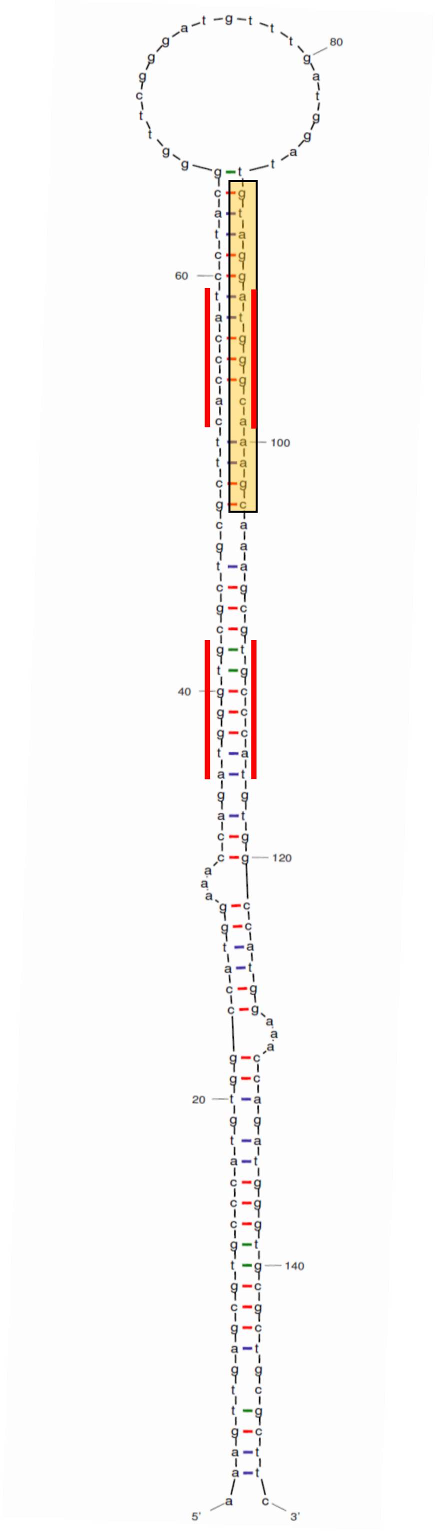

Thioalkalivibrio sp. HL-EbGR7
Position: 1417740..417890
16-mer found adjacent to *tgr7_1317*

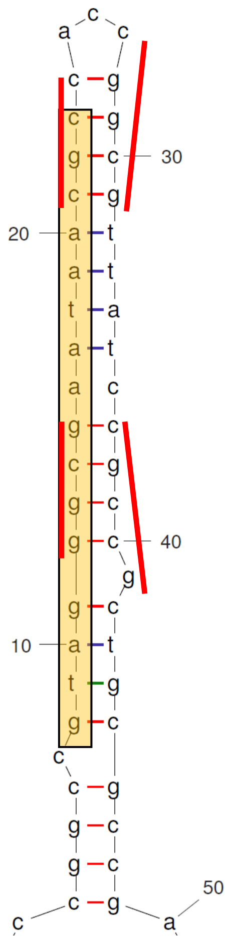

Pseudomonas aeruginosa PA7
Position: 878990..879046
16-mer found adjacent to *pspa7_4226*

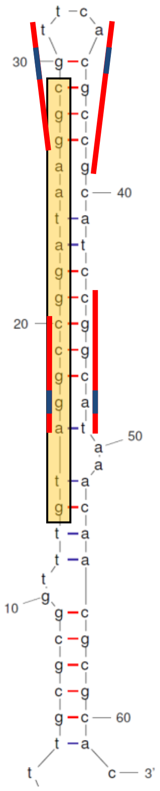

Escherichia coli K-12 DH10B
Position: 868786..868847
16-mer found adjacent to *yafM*

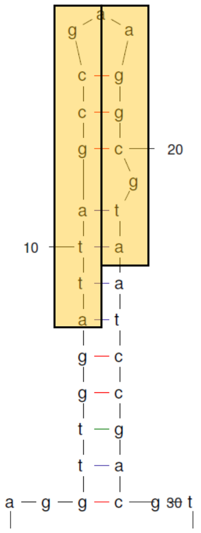

Pseudomonas stutzeri A1501
Position: 1162127_1162158
16-mer found adjacent to *pst_1052*
No typical REPIN formation, only found adjacent to *pst_1052*. Perhaps no dissemination possible.

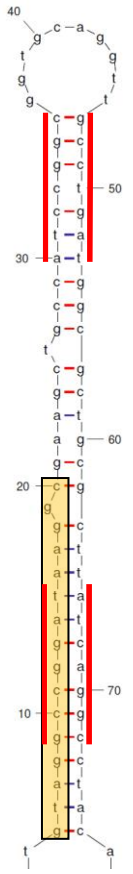

Salmonella enterica serovar Paratyphi A AKU 12601
Position: 298766..298843
16-mer found adjacent to *sspa4070*

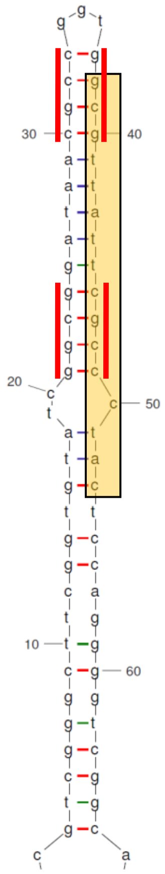

Pseudomonas aeruginosa PAO1 PA1154
Position: 264851..264919
16-mer found adjacent *pa1154*

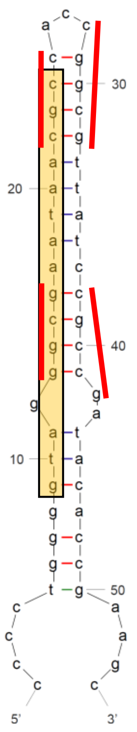

Pseudomonas aeruginosa LESB58
Position: 257386..257439
16-mer found adjacent to *pales_41671*

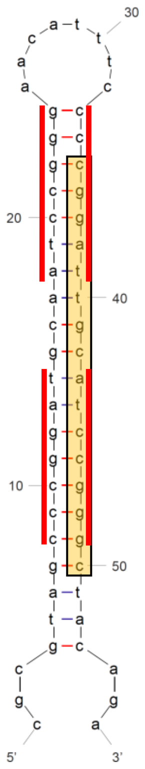

Pseudomonas mendocina ymp
Position: 213188..213243
16-mer found adjacent to *pmen0731*

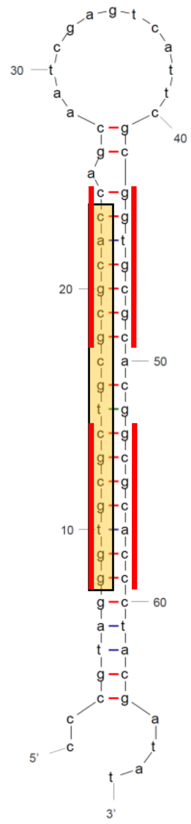

Pseudomonas mendocina ymp
Position: 58161..58228
16-mer found adjacent to *pmen3135*

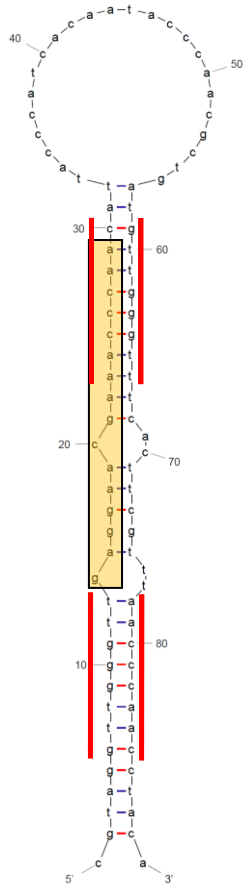

Nostoc punctiforme PCC 73102
Position: 684772400..6847813
16mer found adjacent to *npunF5543*

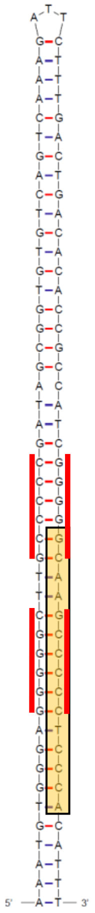

Pseudomonas fluorescens SBW25
GI consensus structure
