# Supplementary material for: Within-Genome Evolution of REPINs: a New Family of Miniature Mobile DNA in Bacteria
Source: PLoS Genet. 2011 Jun 16;7(6):e1002132. doi: 10.1371/journal.pgen.1002132 (PMC3116915; doi:10.1371/journal.pgen.1002132)
Supplement: Table S1 — Dinucleotide frequencies in P. fluorescens Pf0-1 and SBW25. (PDF) [file pgen.1002132.s012.pdf]

Table S1. Dinucleotide frequencies in *P. fluorescens* Pf0-1 and SBW25.

Dinucleotides	Pf0-1	SBW25	Difference to Pf0-1
AA	0.047167435	0.046317477	2%
AC	0.055368846	0.054939965	1%
AG	0.051895004	0.052577167	-1%
AT	0.043838038	0.042889754	2%
CA	0.070204044	0.072515172	-3%
CC	0.077093329	0.081420588	-6%
CG	0.104089616	0.095726792	8%
CT	0.051886151	0.052746299	-2%
GA	0.063428452	0.056363832	11%
GC	0.107768012	0.109277776	-1%
GG	0.076447517	0.081610249	-7%
GT	0.054290163	0.055384886	-2%
TA	0.017469547	0.021527881	-23%
TC	0.063042953	0.056770672	10%
TG	0.069502162	0.072722386	-5%
TT	0.046504227	0.047209105	-2%
