# Supplementary material for: Within-Genome Evolution of REPINs: a New Family of Miniature Mobile DNA in Bacteria
Source: PLoS Genet. 2011 Jun 16;7(6):e1002132. doi: 10.1371/journal.pgen.1002132 (PMC3116915; doi:10.1371/journal.pgen.1002132)
Supplement: Table S2 — Short sequence composition of the non-coding DNA flanking RAYTs. (PDF) [file pgen.1002132.s013.pdf]

Table S2. Short sequence composition of the non-coding DNA flanking RAYTs.

Organism	Name of RAYT homologue	Pos[a]	Most abundant 16-mer in non-coding DNA flanking *RAYTs*	Freq	p-Value[b]	Palindromes (all 16-mers within the palindrome occur at least twice within the genome)
P. fluorescens SBW25	*yafM*	5'	gggcaagcccgctcac	241	2.00E-06	gcggggcaagcccgc
P. fluorescens SBW25	*yafM*	3'	gggcaagcccgctcac	241	2.00E-06	gggctgcttcgcagccc
P. fluorescens SBW25	*pflu2165*	5'	gagggagcttgctccc	208	3.55E-06	gggagcttgctccc
P. fluorescens SBW25	*pflu2165*	3'	gagggagcttgctccc	208	3.55E-06	gggagcttgctccc
P. syringae phaseolicola 1448A	*pspph_4464*	3'	gcaagctcgctcccac	28	4.57E-04	gcgagcaagctcgc
P. syringae phaseolicola 1448A	*pspph_4464*	5'	acgatgcgactttgcc	1	1	none
P. syringae syringae B728a	*psyr_4421*	3'	tcgcgagcaagctcgc	88	4.41E-06	gcgagcaagctcgc
P. syringae syringae B728a	*psyr_4421*	5'	atgtgattgtgatctc	1	1	none
P. syringae syringae B728a	*psyr_4707*	3'	ttcgcgaacaagttcg	201	3.40E-07	gcgaacaagttcgc
P. syringae syringae B728a	*psyr_4707*	5'	gtgtcgttgcgcaatg	1	1	none
P. syringae phaseolicola 1448A	*pspph_5043*	3'	acggcgtgccactgcg	1	1	none
P. syringae phaseolicola 1448A	*pspph_5043*	5'	ggagcggacttgtccg	42	2.40E-04	gcggacttgtccgc
P. syringae tomato DC3000	*pspto_0262*	3'	gcgtgccgctgcgcaa	3	0.004	none
P. syringae tomato DC3000	*pspto_0262*	5'	gagcggacttgtccgc	39	1.81E-04	gcggacttgtccgc
P. fluorescens SBW25	*pflu3939*	5'	gcaagccccctcccac	618	1.54E-07	gggggcaagccccc
P. fluorescens SBW25	*pflu3939*	3'	gtgggagggggcttgc	618	1.54E-07	gggggcttgccccc
P. fluorescens Pf-5	*pfl_3160*	5'	tcgccggcaagccggc	358	1.48E-07	gccggcaagccggc
P. fluorescens Pf-5	*pfl_3160*	3'	tcgccggcaagccggc	358	1.48E-07	gccggcaagccggc
P. entomophila L48	*pseen5170*	5'	gtaggagccagcttgc	95	1.62E-05	gccagcttgctggcg
P. entomophila L48	*pseen5170*	3'	aacactttatccacag	2	0.04	none
T. sp HL-EbGR7	*tgr7_2777*	3'	tcggcctgaaggccga	50	1.59E-05	gtcggcctgaaggccgac
T. sp HL-EbGR7	*tgr7_2777*	5'	tcgggctgaagcccga	80	6.01E-07	gtcgggctgaagcccgac
P. putida W619	*pputw619_5047*	5'	gatcgccggcaagccg	20	2.66E-05	cggcaagccg
P. putida W619	*pputw619_5047*	3'	tcgccggcaagccggc	229	1.79E-07	ggcaagccggcttgcc
P. entomophila L48	*pseen4846*	5'	caaggccgctcccaca	181	4.51E-06	gggccgctgtgcggccc
P. entomophila L48	*pseen4846*	3'	caaggccgctcccaca	181	4.51E-06	gcgacacaaggccgc
P. putida KT2440	*pp_0568*	5'	tgtgggagcggccttg	54	9.65E-06	gcggccttgcgtcgc
P. putida KT2440	*pp_0568*	3'	caaggccgctcccaca	54	9.65E-06	gcgacacaaggccgc

P. putida F1	*pput_0607*	5’	atgagggcgaagccct	2	0.03	tgagggcgaagccctca
P. putida F1	*pput_0607*	3’	caaggccgctcccaca	140	3.50E-06	cgctcccacagggaccgcg
P. putida W619	*pputw619_4597*	3’	gcggccttgtgtcgcg	148	1.07E-06	ggggctgccttgcagccc
P. putida W619	*pputw619_4597*	5’	caaggccgctcctaca	119	3.21E-06	ccgctcctacagggg
P. putida GB1	*pputgb1_0613*	5’	tcgcgacacaaggccg	235	1.71E-07	ggggccgctttgcggccc
P. putida GB1	*pputgb1_0613*	3’	tcgcgacacaaggccg	235	1.71E-07	cgcgacacaaggccgctcctacaggg atcgcg
P. putida GB1	*pputgb1_5236*	5’	aacccgctcccacagg	62	6.32E-06	gcgggtgaacccgc
P. putida GB1	*pputgb1_5236*	3’	tcgcgggtaaacccgc	90	3.07E-06	gcgggtaaacccgc
P. putida KT2440	*pp_5176*	5’	agcccgcgaagaggcc	26	3.20E-05	ctcttcgcgggcgagcccgcgaagag
P. putida KT2440	*pp_5176*	3’	cctgtgggagcgggcg	86	5.25E-06	gcgggcgtgcccgc
P. putida F1	*pput_5083*	5’	cgggcgagcccgcgaa	33	4.07E-05	ggcctcttcgcgggcgagcccgcgaag aggcc
P. putida F1	*pput_5083*	3’	gcccgctcccacaggg	70	1.19E-05	gcgggcatgcccgc
P. putida GB1	*pputgb1_1364*	5’	gccgcccgcgcggcgc	35	1.33E-05	agcgccgcccgcgcggcgct
P. putida GB1	*pputgb1_1364*	3’	gccgcccgcgcggcgc	35	1.33E-05	gcgccgcccgcgcggcgc
P. entomophila L48	*pseen3227*	5’	gcggattcatccgcga	151	6.50E-06	gcggattcatccgc
P. entomophila L48	*pseen3227*	3’	tcgcggatgaatccgc	151	6.50E-06	gcggatgaatccgc
P. putida F1	*pput_3919*	5’	cgggtttacccgcgaa	404	1.75E-07	gcgggtttacccgc
P. putida F1	*pput_3919*	3’	cctcaccccagccgcg	2	0.03	None
P. mendocina ymp	*pmen_3135*	5’	ggtgcgcacggcgcac	198	2.08E-07	ggtgcgcacggcgcacc
P. mendocina ymp	*pmen_3135*	3’	ggtgcgcacggcgcac	198	2.08E-07	ggtgcgcacggcgcacc
T. sp. HL-EbGR7	*tgr7_1317*	5’	gtaggatgggcaaagc	14	1.43E-04	atgggcaaagcgatagcgtgcccat
T. sp. HL-EbGR7	*tgr7_1317*	3’	gtaggatgggcaaagc	14	1.43E-04	atgggcaaagcaacgcgtgcccat
N. punctiforme PCC 73102	*npun_f5543*	5’	gaggaacgaaacccaa	13	4.39E-04	gttgggttgaggaacgaaacccaac
N. punctiforme PCC 73102	*npun_f5543*	3’	atgttgggtttcgttc	13	4.39E-04	gttgggtttcgttcctcaacccaac
P. mendocina ymp	*pmen_0731*	5’	cggattgcatccgggc	93	3.12E-06	cccggattgcatccggg
P. mendocina ymp	*pmen_0731*	3’	cggattgcatccgggc	93	3.12E-06	cccggattgcatccggg
P. stutzeri A1501	*pst_1052*	5’	attagccgaaggcgta	4	0.0029	tggattagccgaaggcgtaatccg
P. stutzeri A1501	*pst_1052*	3’	aaacgacggaagcgcc	2	0.03	None
S. enterica serovar Paratyphi A AKU 12601	*sspa4070*	5’	cgcttaccgggcctac	18	7.57E-06	gcccggtggcgcttcgcttaccgggc
S. enterica serovar Paratyphi A AKU 12601	*sspa4070*	3’	gtaggccggataaggc	57	2.23E-07	aggccggataaggcgt
E coli K-12 DH10B	*yafM*	5’	tgcctgatgcgacgct	77	2.26E-06	gcctgatgcgacgctggcgcgtcttat catgc
E. coli K-12 DH10B	*yafM*	3’	gtaggccggataaggc	106	2.26E-07	aggccggataaggcgt
P. aeruginosa PAO1	*pa1154*	5’	gcgttattcgccctac	30	1.02E-06	gcgttattcgc
P. aeruginosa PAO1	*pa1154*	3’	gcgttattcgccctac	30	1.02E-06	gcgttattcgc
P. aeruginosa PA7	*pspa7_4226*	5’	gtagggcgaataacgc	7	3.15E-04	gcgaataacgc

P. aeruginosa PA7	*pspa7_4226*	3’	gtagggcgaataacgc	7	3.15E-04	gcgaataacgc
P. aeruginosa LESB58	*pales_41671*	5’	gtagggcgaataacgc	26	8.11E-07	gcgaataacgc
P. aeruginosa LESB58	*pales_41671*	3’	gtagggcgaataacgc	26	8.11E-07	gcgaataacgc

All homologues are flanked by at least one 16-mer that is unusually over-represented within the respective genome of the bacterium. In all cases the 16-mer contains or is part of a palindrome or inverted repeat. Letters in red denote complementary base pairs. [a]Denotes whether the 16-mer was found in the extragenic space flanking the RAYT on the 5’ or 3’ side. [b]Proportion of different 16-mers that occur equally or more often than the most abundant 16-mer from the non-coding DNA flanking the RAYT homologue.
