# Supplementary material for: Within-Genome Evolution of REPINs: a New Family of Miniature Mobile DNA in Bacteria
Source: PLoS Genet. 2011 Jun 16;7(6):e1002132. doi: 10.1371/journal.pgen.1002132 (PMC3116915; doi:10.1371/journal.pgen.1002132)
Supplement: Table S4 — Correlation between REPINs and repeat families previously detected in SBW25. (PDF) [file pgen.1002132.s015.pdf]

Table S4. Correlation between REPINs and repeat families previously detected in SBW25.

	GI REPINs[a]	GII REPINs[a]	GIII REPINs[a]
**R0**[b]	152	0	3
**R2**[b]	3	85	51
**others**	37[c]	1	0

[a]Only exact matches of GI, GII and GIII 16-mers were considered when searching for REPINs. [b]Repeat families detected in SBW25 by Silby et al. (2009). [c] The high number of others for GI doublets is attributable to the presence of two different REP orientations within REPINs (TT-AA is found in R0, AA-TT is not).
